# CmoNAC1 in pumpkin rootstocks improves salt tolerance of grafted cucumbers by binding to the promoters of *CmoRBOHD1*, *CmoNCED6*, *CmoAKT1;2* and *CmoHKT1;1* to regulate H_2_O_2_, ABA signaling and K^+^/Na^+^ homeostasis

**DOI:** 10.1093/hr/uhad157

**Published:** 2023-07-25

**Authors:** Yuquan Peng, Haishun Cao, Lvjun Cui, Ying Wang, Lanxing Wei, Shouyu Geng, Li Yang, Yuan Huang, Zhilong Bie

**Affiliations:** National Key Laboratory for Germplasm Innovation & Utilization of Horticultural Crops/College of Horticulture and Forestry Sciences, Huazhong Agricultural University, 430070 Wuhan, China; National Key Laboratory for Germplasm Innovation & Utilization of Horticultural Crops/College of Horticulture and Forestry Sciences, Huazhong Agricultural University, 430070 Wuhan, China; Institute of Facility Agriculture, Guangdong Academy of Agricultural Sciences, 510640 Guangzhou, China; National Key Laboratory for Germplasm Innovation & Utilization of Horticultural Crops/College of Horticulture and Forestry Sciences, Huazhong Agricultural University, 430070 Wuhan, China; National Key Laboratory for Germplasm Innovation & Utilization of Horticultural Crops/College of Horticulture and Forestry Sciences, Huazhong Agricultural University, 430070 Wuhan, China; National Key Laboratory for Germplasm Innovation & Utilization of Horticultural Crops/College of Horticulture and Forestry Sciences, Huazhong Agricultural University, 430070 Wuhan, China; National Key Laboratory for Germplasm Innovation & Utilization of Horticultural Crops/College of Horticulture and Forestry Sciences, Huazhong Agricultural University, 430070 Wuhan, China; National Key Laboratory for Germplasm Innovation & Utilization of Horticultural Crops/College of Horticulture and Forestry Sciences, Huazhong Agricultural University, 430070 Wuhan, China; National Key Laboratory for Germplasm Innovation & Utilization of Horticultural Crops/College of Horticulture and Forestry Sciences, Huazhong Agricultural University, 430070 Wuhan, China; National Key Laboratory for Germplasm Innovation & Utilization of Horticultural Crops/College of Horticulture and Forestry Sciences, Huazhong Agricultural University, 430070 Wuhan, China; Hubei Hongshan Laboratory, 430070 Wuhan, China

## Abstract

The NAC transcription factor is a type of plant-specific transcription factor that can regulate plant salt tolerance, but the underlying mechanism is unclear in grafted vegetables. H_2_O_2_ and ABA in pumpkin rootstocks can be transported to cucumber scion leaves, promoting stomatal closure to improve salt tolerance of grafted cucumbers. Despite these observations, the regulatory mechanism is unknown. Here, our research revealed that CmoNAC1 is a key transcription factor that regulates H_2_O_2_ and ABA signaling in pumpkin roots under salt stress. The function of *CmoNAC1* was analyzed using root transformation and RNA-seq, and we found that pumpkin CmoNAC1 promoted the production of H_2_O_2_ and ABA via *CmoRBOHD1* and *CmoNCED6*, respectively, and regulated K^+^/Na^+^ homeostasis via *CmoAKT1;2*, *CmoHKT1;1*, and *CmoSOS1* to improve salt tolerance of grafted cucumbers. Root knockout of CmoNAC1 resulted in a significant decrease in H_2_O_2_ (52.9% and 32.1%) and ABA (21.8% and 42.7%) content and K^+^/Na^+^ ratio (81.5% and 56.3%) in leaf and roots of grafted cucumber, respectively, while overexpression showed the opposite effect. The root transformation experiment showed that *CmoNCED6* could improve salt tolerance of grafted cucumbers by regulating ABA production and K^+^/Na^+^ homeostasis under salt stress. Finally, we found that CmoNAC1 bound to the promoters of *CmoRBOHD1*, *CmoNCED6*, *CmoAKT1;2*, and *CmoHKT1;1* using yeast one-hybrid, luciferase, and electrophoretic mobility shift assays. In conclusion, pumpkin CmoNAC1 not only binds to the promoters of *CmoRBOHD1* and *CmoNCED6* to regulate the production of H_2_O_2_ and ABA signals in roots, but also binds to the promoters of *CmoAKT1;2* and *CmoHKT1;1* to increase the K^+^/Na^+^ ratio, thus improving salt tolerance of grafted cucumbers.

## Introduction

A significant threat to crop growth and productivity is soil salinity, which affects ~1 billion square hectometers of land worldwide [1, 2]. In agricultural production, unreasonable irrigation and fertilization gradually aggravate soil secondary salinization, which seriously restricts the sustainable development of agriculture [3, 4]. Salt stress leads to Na^+^ toxicity and oxidative damage [5–7]. Na^+^ toxicity is due to the similar hydration radii of Na^+^ and K^+^. Na^+^ competes for the K^+^ binding sites of some enzymes, resulting in reduced enzyme activity and plant metabolic disorders [8, 9]. Under salt stress, excessive formation and accumulation of reactive oxygen species (ROS) cause oxidative damage that leads to membrane lipid peroxidation, malondialdehyde (MDA) accumulation, and increased membrane permeability. [10]. Plants can cope with salt stress by regulating H_2_O_2_ and ABA signaling and ion transport [11, 12]. H_2_O_2_ and ABA can promote stomatal closure in response to salt stress [13, 14]. The regulatory mechanisms of ion transport mainly include: (1) increased *SOS1* and *SOS2* expression promoting Na^+^ efflux [15, 16]; (2) upregulated *NHX1* expression causing the storage of excessive Na^+^ in vacuoles [17, 18]; (3) increased *HKT1* expression leading to the inhibition of Na^+^ translocation from the root to the shoot [19]; and (4) upregulated expression of K^+^ transporter genes such as *HAK5* and *AKT1*, increasing K^+^ absorption [20, 21].

NAC transcription factors are plant-specific. In recent years, NAC transcription factors have become a research hotspot in response to salt stress [22–25]. At the N-terminus, NAC transcription factors possess a highly conserved NAC domain of ~170 amino acids. This domain directly binds to the promoters of target genes, forming a helix–turn–helix structure that can either activate or inhibit transcription. Conversely, the C-terminal exhibits a high degree of variability and serves as a transcriptional regulation region with an intrinsically disordered structure, which can be influenced by external stimuli [26, 27]. In the promoters of genes targeted by NAC transcription factors, CGTG, CGTA, or CACG motifs are commonly observed [28]. These motifs serve as recognition sequences for NAC transcription factors [29, 30]. Although the promoter binding sites of NAC transcription factors are conserved, the promoter regions of the same genes in different species are not conserved, leading to differences in the regulatory mechanisms of NAC transcription factors in different species under salt stress. The reported mechanisms of NAC transcription factors regulating salt stress mainly include: (1) binding to gene promoters such as *RBOH* and *DREB* to regulate the production of H_2_O_2_ and ABA, respectively [31, 32]; (2) promoting the expression of K^+^/Na^+^ transport genes to improve the K^+^/Na^+^ ratio [33–35]; (3) enhancing the activity of antioxidant enzymes, such as superoxide dismutase (SOD), peroxidase (POD), and catalase (CAT) [34]; and (4) interacting with transcription factors to form protein complexes that synergistically activate the expression of the E3 ubiquitin ligase *MREL57* gene to enhance its salt tolerance [36]. Our previous study revealed that overexpression of pumpkin *CmoNAC1* improved salt tolerance in *Arabidopsis thaliana* [37], but the function and regulatory mechanism of the NAC transcription factor in grafted cucumbers under salt stress has not been reported.

Grafting is an important agronomic measure that has been widely used in the production of cucurbitaceous and Solanaceae vegetables [38, 39]. Pumpkin, a popular cucurbit crop with a substantial industrial value of 1.6 billion dollars (https://www.fao.org/faostat/en/#search/pumpkin, 2021), is frequently used as a rootstock to enhance cucumber’s salt tolerance. Moreover, grafting is also an ideal method to study signal communication between plant shoots and roots [40]. Pumpkin and cucumber are typical cucurbit crops with well-developed vascular tissues, which are ideal vectors for the study of long-distance signal transduction between rootstocks and scions [41]. Several primary mechanisms contribute to the enhancement of salt tolerance in vegetable crops through grafting: (1) synthesis of H_2_O_2_ and ABA in rootstocks, followed by their transport to scions to promote stomatal closure [42, 43]; (2) enhancing *CsHAK5;3* expression in scion leaves to improve K^+^ absorption [20]; (3) regulation of CmHKT1 localization to the plasma membrane through CmCNIH1, thereby restricting Na^+^ transfer to scions [44]; (4) enhancement of the SOD and POD enzyme activity in scion leaves to mitigate oxidative damage [36]; and (5) stimulating the accumulation of osmotic regulators like proline to reduce water loss in scion leaves [45]. Our previous study showed that in grafted cucumbers, H_2_O_2_ (*CmoRBOHD1*-mediated) and ABA in pumpkin rootstocks were upregulated and transported to scions, which promoted stomatal closure at 3 h of 75 mM NaCl treatment and thus enhanced salt tolerance [42, 43, 46]. However, the key transcription factors that co-regulate H_2_O_2_ and ABA signaling in pumpkin rootstocks have not yet been identified.

In this study, we found that CmoNAC1 is a key transcription factor that regulates H_2_O_2_ and ABA signaling in pumpkin rootstocks under salt stress through promoter and transcriptional analyses. Root transformation and RNA-seq techniques revealed the function of CmoNAC1 and CmoNCED6 in pumpkin rootstocks to improve salt tolerance of grafted cucumbers. Next, the binding effect of CmoNAC1 on the *CmoRBOHD1*, *CmoNCED6*, *CmoAKT1;2*, and *CmoHKT1;1* promoters was analyzed using the yeast one-hybrid assay (Y1H), luciferase (LUC) assay, and electrophoretic mobility shift assay (EMSA). By uncovering the function and mechanism of pumpkin CmoNAC1 in improving salt tolerance in grafted cucumbers, our study provides valuable insights into the broader understanding of plant salt tolerance. These findings serve as a valuable reference for future molecular breeding efforts aimed at developing salt-tolerant pumpkin rootstocks.

## Results

### CmoNAC1 is a key transcription factor regulating H_2_O_2_ and ABA signaling in pumpkin rootstocks under salt stress

**Figure 1:**
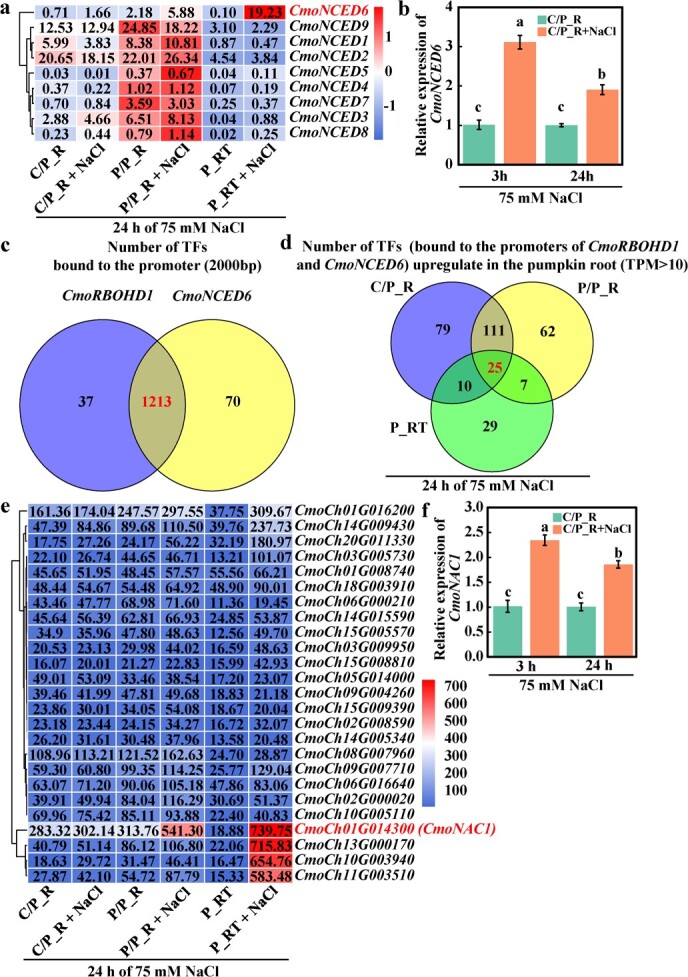
Screening of key transcription factors (TFs) co-regulating H_2_O_2_ and ABA signaling in grafted cucumber under salt stress. **a** Heat map of pumpkin *NCED* gene expression. **b** Relative expression of *CmoNCED6*. **c**, **d** Number of TFs bound to the promoters of *CmoRBOHD1* and *CmoNCED6* and upregulated in the pumpkin root (TPM > 10). **e** Heat map of upregulated TF expression. **f** Relative expression of *CmoNAC1* in the root of C/P. Mean ± standard error (*n* = 3). Different lowercase letters indicate significant differences among different treatments at the *P* < .05 level.

In pumpkin rootstocks, respiratory burst oxidase homolog D1 (*CmoRBOHD1*) plays a major role in H_2_O_2_ signaling under salt stress, whereas the key gene for ABA synthesis has not been identified. The enzyme 9-*cis*-epoxycarotenoid dioxygenase (NCED) is essential for the conversion of neoxanthin to xanthoxin, a key precursor in the ABA biosynthesis pathway. Firstly, we searched for homologs of *NCED* and identified nine *NCED* genes in pumpkin (Supplementary Data Fig. S1). The expression levels of these *NCED* genes were analyzed, and it was found that *CmoNCED6* was the most induced in C/P (cucumber scion/pumpkin rootstock), P/P (pumpkin scion/pumpkin rootstock) and P_RT (pumpkin root tip) after salt treatment for 24 h, which showed 1.2­, 1.7­ and 182.3­ folds induction, respectively (Fig. 1a). The expression level of *CmoNCED6* was further analyzed by qRT–PCR. Compared with 0 mM NaCl, the expression level of *CmoNCED6* in pumpkin rootstocks treated with salt was upregulated by 2.1 and 0.9 times at 3 and 24 h, respectively (Fig. 1b). These results indicated that *CmoNCED6* was the key gene for ABA synthesis in pumpkin rootstocks under salt stress.

To identify the key transcription factors that regulate the production of H_2_O_2_ and ABA in pumpkin rootstocks under salt stress, the promoters of *CmoRBOHD1* and *CmoNCED6* were analyzed, and 1213 transcription factors were found to bind to their promoters (Fig. 1c). The expression levels of these transcription factors were analyzed. We found that 25 transcription factors with TPM (transcripts per million) >10 and upregulated expression in C/P, P/P, and P_RT after 24 h salt treatment. Notably, *CmoNAC1* exhibited the highest level of expression among these transcription factors (Fig. 1d and e). qRT–PCR results showed that, compared with the control (0 mM NaCl), the expression level of *CmoNAC1* in pumpkin rootstocks was upregulated by 1.3 and 0.8 times at 3 and 24 h, respectively (Fig. 1f). These results suggest that CmoNAC1 is likely an important transcription factor regulating H_2_O_2_ and ABA signaling in pumpkin rootstock under salt stress.

### *CmoNAC1* improves salt tolerance of grafted cucumbers by regulating K^+^/Na^+^ homeostasis

**Figure 2:**
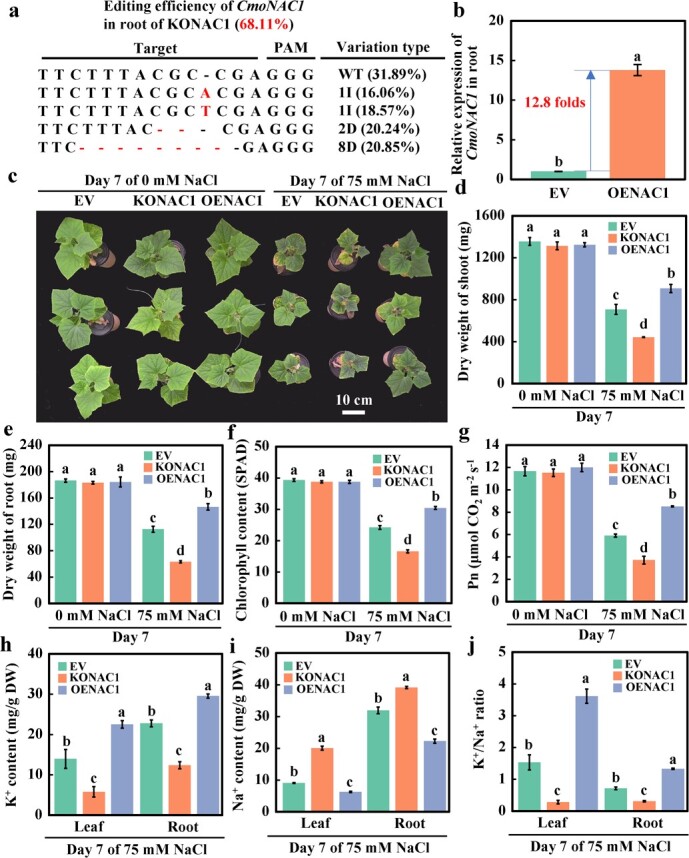
Effects of salinity on the growth of grafted cucumber with root knockout and overexpression of *CmoNAC1*. **a** Editing efficiency of *CmoNAC1* by Hi-TOM sequencing. **b** Quantitative PCR analysis of relative expression of *CmoNAC1* in the roots with overexpressed *CmoNAC1*. **c**–**e** Phenotypic performance, shoot, and root dry weight. **f**, **g** Relative chlorophyll content and Pn of leaf in KONAC1 and OENAC1. **h**–**j** K^+^ content, Na^+^ content, and K^+^/Na^+^ ratio. Mean ± standard error (*n* = 3). Different lowercase letters indicate significant differences among different treatments at the *P* < .05 level.

To further investigate the function of CmoNAC1 in enhancing the salt resistance of grafted cucumbers, we grafted cucumbers onto roots transfected with empty vector (EV), *CmoNAC1* knockout construct (KONAC1), and *CmoNAC1* overexpression construct (OENAC1) by root transformation. Hi-TOM analysis showed that the gene editing efficiency of *CmoNAC1* in the KONAC1 root was 68.11% (Fig. 2a). Compared with EV, the *CmoNAC1* expression level in OENAC1 roots was increased 12.8 times (Fig. 2b). The salt tolerance of EV, KONAC1, and OENAC1 was analyzed. Compared with EV, the phenotype (Fig. 2c–g; Supplementary Data Fig. S2), damage index (Supplementary Data Fig. S3), and photosynthetic index (Supplementary Data Fig. S4) of KONAC1 and OENAC1 did not change significantly at day 7 without NaCl treatment, indicating that the knockout and overexpression of *CmoNAC1* had no significant effect on the phenotype of grafted cucumbers. However, KONAC1 exhibited reduced biomass accumulation (shoot and root dry weight decreased by 37.6% and 43.9%, respectively) with a higher degree of damage after 7 days of salt treatment compared with EV (Fig. 2c–e; Supplementary Data Figs S2a–d andS3a–d), and a lower photosynthetic capacity [chlorophyll content and net photosynthetic rate (Pn) decreased by 31.4% and 37.0%, respectively] (Fig. 2f and g; Supplementary Data Fig. S4a–e). In contrast, the growth of OENAC1 was enhanced (shoot and root dry weight increased by 28.2% and 30.1%, respectively) with a lower degree of damage (Fig. 2c–e; Supplementary Data Figs S2a–d and S3a–d), and a higher photosynthetic capacity (chlorophyll content and Pn increased by 25.8% and 44.2%, respectively) (Fig. 2f and g, Supplementary Data Fig. S4a–e). These results indicated that *CmoNAC1* in pumpkin rootstocks was significant in modulating salt tolerance in grafted cucumbers*.* Next, the K^+^/Na^+^ content was analyzed on day 7 of salt stress. Compared with EV, in leaves and roots of KONAC1 the K^+^ content decreased by 58.6% and 45.7%, respectively, and the Na^+^ content increased by 121.1% and 22.6%. Consequently, the K^+^/Na^+^ ratio in the leaves and roots was reduced by 81.5% and 56.3%, respectively. Conversely, in OENAC1 the K^+^ content increased by 61.4% and 29.8%, while the Na^+^ content decreased by 31.1% and 30.3% in the leaves and roots, respectively. This resulted in an increase in K^+^/Na^+^ ratio of 135.9% and 85.6% in the leaves and roots of OENAC1 (Fig. 2h and j). These findings demonstrate that CmoNAC1 in pumpkin rootstocks plays a significant role in enhancing salt tolerance in grafted cucumbers.

### *CmoNAC1* regulates H_2_O_2_ and ABA signaling in grafted cucumbers, thereby mediating stomatal closure under salt stress

**Figure 3:**
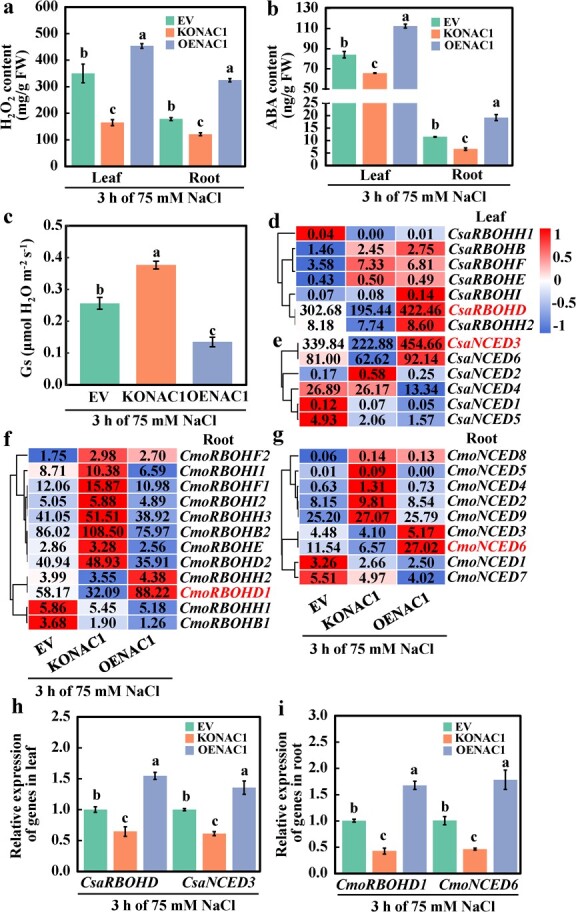
Effects of salinity on H_2_O_2_ and ABA content and signaling in cucumber scion leaves grafted on the *CmoNAC1* knockout or overexpression rootstocks. **a**, **b** H_2_O_2_ and ABA content. **c***G*_s_ of the leaf. **d**–**g** Heat maps of *RBOHs*/*NCED* gene expression. **h**, **i** Relative expression of *CsaRBOHD*/*CsaNCED3* and *CmoRBOHD1*/*CmoNCED6*. Mean ± standard error (*n* = 3). Different lowercase letters indicate significant differences among different treatments at the *P* < .05 level.

To verify the regulatory effect of *CmoNAC1* on H_2_O_2_ and ABA signaling in grafted cucumbers under salt stress, the H_2_O_2_ and ABA contents and stomatal conductance of leaves in EV, KONAC1, and OENAC1 were analyzed at 3 h after salt treatment. Compared with EV, in leaves and roots of KONAC1 the H_2_O_2_ content decreased by 52.9% and 32.1%, respectively, the ABA content decreased by 21.8% and 42.7%, respectively, and stomatal conductance (*G*_s_) of leaves increased by 47.0%. In leaves and roots of OENAC1 the H_2_O_2_ content increased by 29.7% and 82.1%, respectively, the ABA content increased by 33.9% and 67.6%, respectively, and *G*_s_ of leaves decreased by 47.4% (Fig. 3a–c). These findings suggest that CmoNAC1 controls H_2_O_2_ and ABA production in grafted cucumber leaves and roots to promote stomatal closure under salt stress.

To further elucidate the effects of *CmoNAC1* knockout and overexpression in the roots on the expression levels of H_2_O_2_- and ABA-related genes in grafted cucumbers, RNA-seq was conducted on the leaves and roots of EV, KONAC1, and OENAC1 after 3 h salt treatment. Principal component analysis of the transcriptome data revealed that all three samples of leaves and roots were located within 95% confidence ellipses, indicating reproducibility (Supplementary Data Fig. S5a and b). Additionally, the transcriptome data was validated using qRT–PCR, and the results were consistent between RNA-seq and qRT–PCR (Supplementary Data Fig. S5c). Moreover, the correlation coefficient *R*^2^ was 0.91 (Supplementary Data Fig. S5d), indicating the precision and reliability of the transcriptome data. Compared with KONAC1, the numbers of upregulated genes in the leaves and roots of OENAC1 were increased by 79.6% and 24.1%, respectively, and the numbers of downregulated genes were increased by 31.2% and 59.5%, respectively (Supplementary Data Fig. S6a and b). The results indicate that *CmoNAC1* is required for transcriptional reprogramming in response to salt stress.

Root knockout and overexpression of *CmoNAC1* altered expression of different genes in grafted cucumber under salt stress. To better understand the key genes regulated by *CmoNAC1* in response to salt stress in grafted cucumber, we performed Gene Ontology (GO) enrichment analysis on the differentially expressed genes identified in KONAC1 and OENAC1. Venn diagram analysis revealed that among the common differentially expressed genes of KONAC1 and OENAC1, 637 and 629 genes were found in leaves and roots, respectively (Supplementary Data Fig. S6c and d). GO enrichment of these genes showed that they were enriched in ion transport and redox activities, suggesting that CmoNAC1 regulates ion transport and redox functions in grafted cucumber leaves and roots under salt stress (Supplementary Data Fig. S6e and f). We analyzed the H_2_O_2_ synthesis gene *RBOH* and identified 12 and 7 *RBOH* genes in pumpkin and cucumber, respectively (Supplementary Data Fig. S7a). In addition, six *NCED* genes were identified in cucumber (Supplementary Data Fig. S7b). Transcriptome data were employed to investigate the expression levels of *RBOH* and *NCED* genes in leaves and roots. *CmoNAC1* knockout resulted in the significant downregulation of *CsaRBOHD*, *CsaRBOHH2*, *CsaNCED3*, and *CsaNCED6* in grafted cucumber leaves and *CmoRBOHD1*, *CmoRBOHH2*, *CmoNCED3*, and *CmoNCED6* in roots under salt stress, while overexpression showed the opposite effect. Among the eight genes, *CsaRBOHD*, *CsaNCED3*, *CmoRBOHD1*, and *CmoNCED6* had significantly higher expression levels (Fig. 3d–g). In order to further clarify the regulatory effect of CmoNAC1 on *RBOH* and *NCED* genes in pumpkin roots, we analyzed the expression of different *RBOH* and *NCED* genes in pumpkin roots with knocked-out and overexpressed *CmoNAC1* under 75 mM NaCl treatment at 3 h (Supplementary Data Fig. S7c and d). We found that, among all *RBOH* genes, only *CmoRBOHD1* demonstrated a significant opposite change compared with the control (EV) in KONAC1 and OENAC1. Similarly, among all *NCED* genes, only *CmoNCED6* showed a significant opposite change in KONAC1 and OENAC1 compared with EV. Therefore, our result indicates that CmoNAC1 specifically regulates the expression of *CmoRBOHD1* and *CmoNCED6* at 3 h under 75 mM NaCl treatment in pumpkin roots. Next, qRT–PCR was further used to verify their expression levels. Compared with EV, *CsaRBOHD* and *CsaNCED3* in KONAC1 leaves were downregulated by 35.4% and 38.9%, respectively, and *CmoRBOHD1* and *CmoNCED6* in roots were downregulated by 57.2% and 54.0%, respectively. *CsaRBOHD* and *CsaNCED3* in OENAC1 leaves increased by 54.4% and 35.4%, respectively, and *CmoRBOHD1* and *CmoNCED6* in roots increased by 67.5% and 77.5%, respectively (Fig. 3h and i). These results indicated that CmoNAC1 affected the production of H_2_O_2_ and ABA by regulating the expressions of *CsaRBOHD* and *CsaNCED3* in grafted cucumber leaves and *CmoRBOHD1* and *CmoNCED6* in roots.

### *CmoNCED6* improves salt tolerance of grafted cucumbers by enhancing ABA signaling-mediated stomatal closure and K^+^/Na^+^ homeostasis

**Figure 4:**
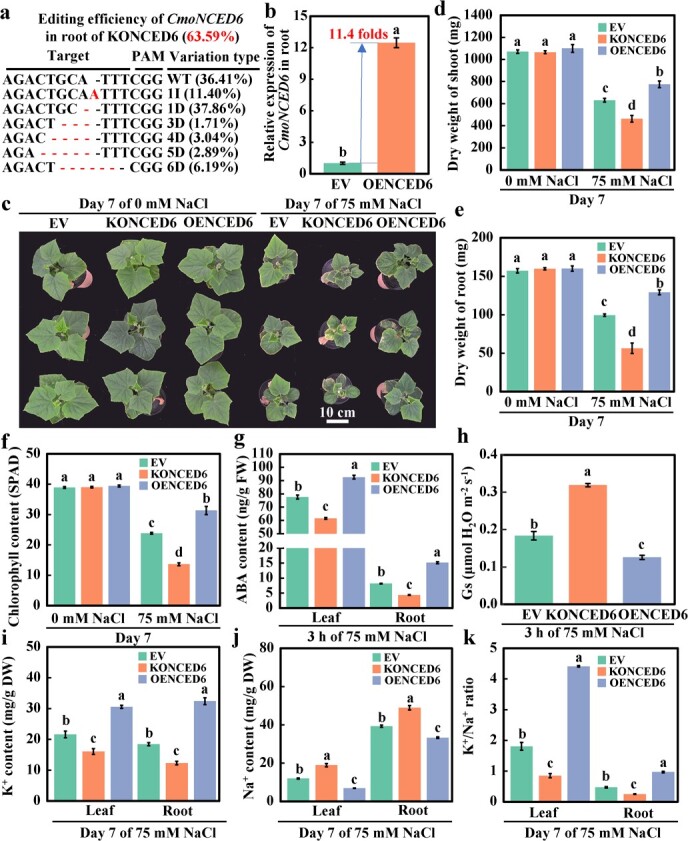
Effects of salinity on the growth of grafted cucumber with root knockout and overexpression of *CmoNCED6*. **a** Heat map of *NCED* genes expression in the root of pumpkin under salt stress. **b** Editing efficiency of *CmoNCED6* by Hi-TOM sequencing. **c** Relative expression of *CmoNCED6* in roots with overexpressed *CmoNCED6*. **d**–**f** Phenotypic performance and shoot and root dry weight. **g** ABA content. **h***G*_s_ of the leaf. **i**–**k** K^+^ content, Na^+^ content, and K^+^/Na^+^ ratio. Mean ± standard error (*n* = 3). Different lowercase letters indicate significant differences among different treatments at the *P* < .05 level.

To verify the function of *CmoNCED6* in the salt tolerance of grafted cucumbers, we grafted cucumbers onto the root transfected by the EV, *CmoNCED6* knockout vector (KONCED6), and *CmoNCED6* overexpression vector (OENCED6) using root transformation. Hi-TOM analysis showed that the gene editing efficiency of *CmoNCED6* in the KONCED6 root was 63.59% (Fig. 4a). qRT–PCR showed that, compared with EV, *CmoNCED6* in the root of OENCED6 was upregulated by 11.4 times (Fig. 4b). The salt tolerance of EV, KONCED6, and OENCED6 was analyzed. It was found that the phenotype (Fig 4c–e; Supplementary Data Fig. S8a–d), damage index (Supplementary Data Fig. S9a–d), and photosynthetic index (Fig. 4f and g; Supplementary Data Fig. S10a–e) of KONCED6 and OENCED6 did not change significantly compared with EV at 7 days of 0 mM NaCl, indicating that knockout and overexpression of *CmoNCED6* in rootstocks had no significant effect on the phenotype of grafted cucumber scions. However, compared with EV after 7 days of salt treatment, KONCED6 showed reduced biomass accumulation (shoot and root dry weight decreased by 26.6% and 28.9%, respectively) (Fig. 4c–e; Supplementary Data Fig. S8a–d) with a higher degree of damage (Supplementary Data Fig. S9a–d), and a lower photosynthetic capacity (chlorophyll content decreased by 42.9%) (Fig. 4f; Supplementary Data Fig. S10a–f). In contrast, in OENCED6 the phenotype was better (the shoot and root dry weight increased by 22.8% and 23.8%, respectively) (Fig. 4c–e; Supplementary Data Fig. S8a–d) with a lower degree of damage (Supplementary Data Fig. S9a–d) and a higher photosynthetic capacity (chlorophyll content increased by 31.4%) (Fig. 4f; Supplementary Data Fig. S10a–f). These results indicated that *CmoNCED6* was the key gene regulating the salt tolerance of grafted cucumbers. To further verify the function of *CmoNCED6* in regulating ABA signaling and stomatal closure of grafted cucumbers under salt stress, the ABA content and stomatal conductance in EV, KONCED6, and OENCED6 were analyzed after 3 h of salt treatment. In leaves and roots of KONCED6, ABA content decreased by 20.6% and 46.6%, respectively, and *G*_s_ of leaves increased by 74.0%. In leaves and roots of OENCED6, ABA content increased by 19.2% and 84.7%, respectively, and *G*_s_ of leaves decreased by 31.5% (Fig. 4g and h). These results indicated that *CmoNCED6* could regulate ABA synthesis and promote stomatal closure in grafted cucumbers under salt stress.

The K^+^ and Na^+^ contents in EV, KONCED6, and OENCED6 were analyzed after 7 days of salt treatment. In comparison with EV, in the leaves and roots of KONCED6 the K^+^ content decreased by 25.7% and 33.0%, the Na^+^ content increased by 57.9% and 24.4%, and the K^+^/Na^+^ ratio decreased by 53.0% and 46.8%, respectively. On the other hand, in the leaves and roots of OENCED6, the K^+^ content increased by 41.4% and 75.9%, the Na^+^ content decreased by 42.4% and 15.3%, and the K^+^/Na^+^ ratio increased by 144.1% and 106.4%, respectively (Fig. 4i–k). These findings demonstrate that *CmoNCED6* enhances the salt tolerance of grafted cucumbers by increasing the K^+^/Na^+^ ratio under salt stress.

### CmoNAC1 binds to the promoters of *CmoRBOHD1* and *CmoNCED6*

**Figure 5:**
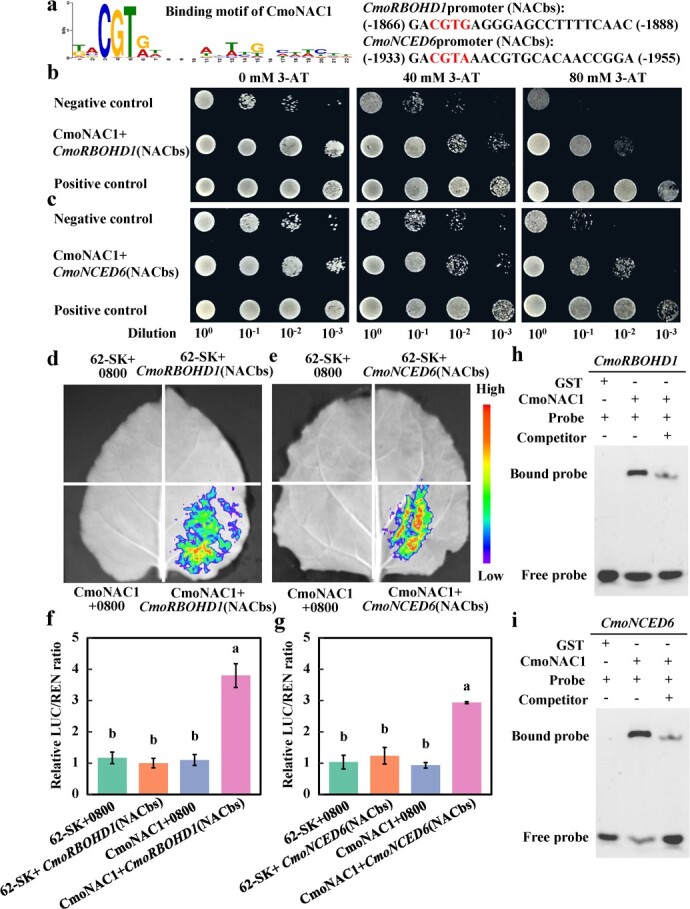
Analysis of binding sites of CmoNAC1 to the *CmoRBOHD1* and *CmoNCED6* promoters. **a** Binding site of CmoNAC1 to the *CmoRBOHD1* and *CmoNCED6* promoter. **b*,* c** Y1H assay, **d**–**g** LUC assay, and (**h**, **i**) EMSA assay identified interactions of CmoNAC1 with the promoters of *CmoRBOHD1* and *CmoNCED6*. Mean ± standard error (*n* = 3). Different lowercase letters indicate significant differences among different treatments at the *P* < .05 level.

To clarify the regulatory mechanism of CmoNAC1 in the expression of *CmoRBOHD1* and *CmoNCED6*, the promoters of *CmoRBOHD1* and *CmoNCED6* were analyzed. We found that 1866–1888 and 1933–1955 bp before ATG of *CmoRBOHD1* and *CmoNCED6,* respectively, were NAC binding sites (NACbs) (Fig. 5a). The binding of CmoNAC1 to *CmoRBOHD1* and *CmoNCED6* NACbs was further analyzed using Y1H. When diluted 10^−2^ at 80 mM 3-AT, the yeast in the negative control did not grow, but the yeast co-transformed with *CmoNAC1* and *CmoRBOHD1/CmoNCED6* NACbs could grow (Fig. 5b and c). This indicated that CmoNAC1 could bind to the NACbs of *CmoRBOHD1* and *CmoNCED6*. LUC was used to verify the binding of CmoNAC1 to the NACbs of *CmoRBOHD1* and *CmoNCED6*. It was found that, compared with the control, the *Nicotiana benthamiana* leaves co-transfected with *CmoNAC1* and the NACbs of *CmoRBOHD1*/*CmoNCED6* showed visible fluorescence, and their relative LUC activities were significantly increased, which also indicated that CmoNAC1 could bind with the NACbs of *CmoRBOHD1* and *CmoNCED6* (Fig. 5d–g). Finally, EMSA was used to directly verify the binding of CmoNAC1 to the NACbs of *CmoRBOHD1* and *CmoNCED6*. The NACbs of *CmoRBOHD1* and *CmoNCED6* labeled with 6-FAM were used as probes. Compared with the addition of glutathione-*S*-transferase (GST) protein, after adding CmoNAC1 protein, obvious bands appeared in both of them. After the addition of unlabeled competing probes, the bands became significantly weaker (Fig. 5h and i), indicating that CmoNAC1 could bind to the NACbs of *CmoRBOHD1* and *CmoNCED6*.

### CmoNAC1 regulates K^+^/Na^+^ homeostasis in grafted cucumbers by binding to the promoters of *CmoAKT1;2* and *CmoHKT1;1* under salt stress

**Figure 6:**
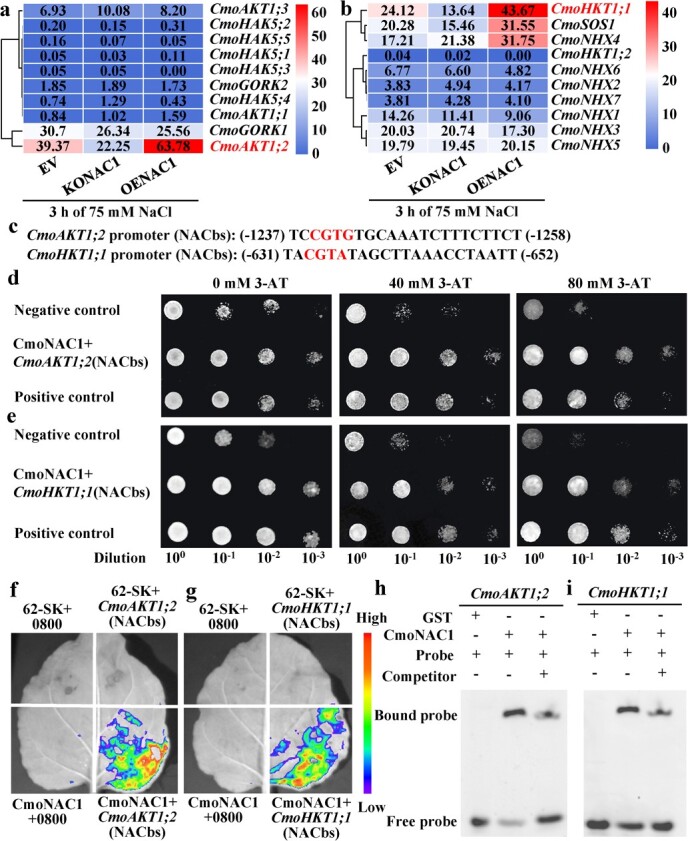
Analysis of binding sites of CmoNAC1 to the K^+^/Na^+^ transport-related gene promoters. **a**, **b** Heat map of Na^+^/K^+^ transport-related gene expression. **c** Binding site of CmoNAC1 to the *CmoAKT1;2* and *CmoHKT1;1* promoters. **d**, **e** Y1H assay identifying interactions of CmoNAC1 with the promoters of *CmoAKT1;2* and *CmoHKT1;1*. **f**, **g** LUC assay identifying interactions of CmoNAC1 with the promoters of *CmoAKT1;2* and *CmoHKT1;1*. **h**, **i** EMSA assay identifying interactions of CmoNAC1 with the promoters of *CmoAKT1;2* and *CmoHKT1;1*.

To clarify the regulatory effect of CmoNAC1 on K^+^/Na^+^ transporters in pumpkin under salt stress, the expression levels of K^+^/Na^+^ transporters in EV, KONAC1, and OENAC1 were analyzed. CmoNAC1 knockout in rootstocks significantly decreased the expression levels of K^+^ transporter *CmoAKT1;2* and Na^+^ transporter *CmoHKT1;1* and *CmoSOS1*, while overexpression showed the opposite effect (Fig. 6a and b). NACbs were found in the promoters of *CmoAKT1;2* and *CmoHKT1;1* but not *CmoSOS1* (Fig. 6c). Therefore, qRT–PCR was used to analyze the expression of *CmoAKT1;2* and *CmoHKT1;1*. Compared with EV, the expression levels of *CmoAKT1;2* and *CmoHKT1;1* in KONAC1 root decreased by 74.7 and 69.8%, and in OENAC1 they increased by 171.3 and 78.2%, respectively (Supplementary Data Fig. S11). The binding of CmoNAC1 to *CmoAKT1;2* and *CmoHKT1;1* NACbs was analyzed using Y1H. When diluted 10^2^ and 10^3^ at 80 mM 3-AT, the yeast in the negative control did not grow, whereas the yeast co-transformed with *CmoNAC1* and *CmoAKT1;2/CmoHKT1;1* NACbs could grow (Fig. 6d and e). This indicates that CmoNAC1 can bind to the NACbs of *CmoAKT1;2* and *CmoHKT1;1*. LUC was further used to verify the binding of CmoNAC1 to the NACbs of *CmoAKT1;2* and *CmoHKT1;1*. Compared with the control, *N. benthamiana* leaves co-transfected with *CmoNAC1* and *CmoAKT1;2/CmoHKT1;1* NACbs showed visible fluorescence (Fig. 6f and g), and their relative LUC activity increased significantly (Supplementary Data Fig. S12a and b). This indicates that CmoNAC1 can bind to the NACbs of *CmoAKT1;2* and *CmoHKT1;1*. Finally, EMSA was used to directly verify the binding of CmoNAC1 to the NACbs of *CmoAKT1;2* and *CmoHKT1;1* when 6-FAM-labeled *CmoAKT1;2* and *CmoHKT1;1* NACbs were used as probes. When CmoNAC1 protein was added, obvious bands appeared in both of them. After the addition of unlabeled competing probes, the bands became significantly weaker (Fig. 6h and i), indicating that CmoNAC1 could bind to the NACbs of *CmoAKT1;2* and *CmoHKT1;1*.

## Discussion

### *CmoNAC1* is a key transcription factor positively regulating salt tolerance in grafted cucumbers

NAC transcription factors have been reported to regulate salt tolerance in various crops. In tomatoes, overexpression of NAC transcription factors *SlTAF1* and *SlNAC1* can significantly increase salt tolerance [23, 47]. In cowpeas, overexpression of *VuNAC1/2* significantly improves salt tolerance, while silencing *VuNAC1/2* significantly reduces salt tolerance [35]. In soybeans, overexpression of *GmNAC181* significantly improves salt tolerance [48]. In rice, knocking out *OsNAC3* and *OsNAC45* significantly reduces salt tolerance [49, 50]. In *A. thaliana*, overexpression of pumpkin *CmoNAC1* can improve salt tolerance [37].

In this study, *CmoNAC1* knockout in rootstocks significantly reduced the salt tolerance of grafted cucumbers, resulting in a significant decrease in the shoot and root dry weight, a higher degree of damage, and a decrease in photosynthetic capacity (Fig. 2c–g; Supplementary Data Figs S2–S4), which was similar to the results in cowpea [35] and rice [49, 50]. Overexpression of *CmoNAC1* in roots significantly improved the salt tolerance of grafted cucumbers, increased the dry weight of shoots and roots, reduced the degree of damage, and enhanced photosynthetic capacity (Fig. 2c–g; Supplementary Data Figs S2–S4), which is similar to the results in tomatoes [23, 47], cowpeas [35], soybeans [48], and *Arabidopsis* [37]. In all of these studies, only gene editing or overexpression techniques were used to study the function of the NAC transcription factor in salt tolerance. However, in this study, by combining root transformation technology with grafting, it was found that pumpkin *CmoNAC1* is a key transcription factor that positively regulates salt tolerance of grafted cucumbers.

**Figure 7:**
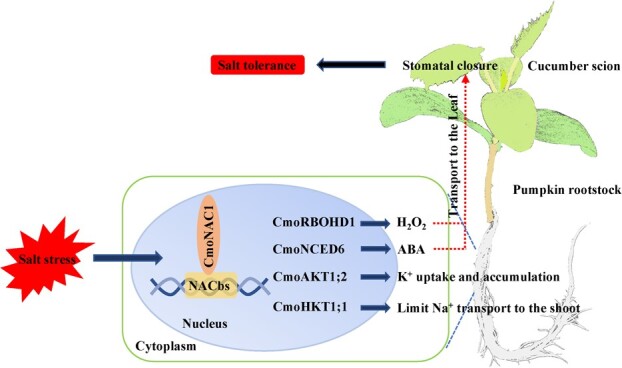
Mechanism by which CmoNAC1 positively regulates salt tolerance in grafted cucumber. Under salt stress, CmoNAC1 can bind with NACbs on the promoters of *CmoRBOHD1* and *CmoNCED6* to regulate the generation of H_2_O_2_ and ABA signals in roots and promote stomatal closure through transport to the shoot; it can also bind with NACbs on the promoters of *CmoAKT1;2* and *CmoHKT1;1* to promote the absorption and accumulation of K^+^ and limit the transport of Na^+^ to the shoot.

### CmoNAC1 is a convergent point of H_2_O_2_ and ABA signaling by binding to the promoters of *CmoRBOHD1* and *CmoNCED6* in response to salt stress in grafted cucumbers

NAC transcription factors can regulate the production of H_2_O_2_ and ABA by binding to the promoters of genes such as *RBOH*, *AREB*, *DREB*, and *NCED*, thus affecting plant salt tolerance. In soybeans, the NAC-like transcription factor GmSIN1 binds to the [CA][CT][TAG]CC[AC]CC[AGC] motif in the promoters of *GmRbohB* and *GmNCED3*s. This interaction facilitates the positive regulation of gene expression, leading to the rapid accumulation of H_2_O_2_ and ABA, which in turn regulates root growth under salt stress [32]. In apples, the binding site of NAC transcription factor SND1 is [T/A]NN[C/T][T/C/G]TNNNNNNNA[A/C]GN[A/C/T][A/T]. SND1 binds to the promoters of *MdDREB2A*, *MdAREB1A*, and *MdAREB1B*. This binding activates their transcription, thereby inducing the transduction of ABA signals and improving salt tolerance [31]. In sweet potatoes, under salt stress, IbNAC3 binds to the CGT[G/A] motif in the promoter of *ERA1*, a critical negative regulator of ABA signaling, and increases its expression to regulate ABA signaling [51]. In *A. thaliana*, pumpkin CmoNAC1 promotes *AtNCED3*-mediated ABA signaling to enhance salt tolerance [37]. Moreover, H_2_O_2_ and ABA are important early signals to improve the salt tolerance of grafted vegetable crops [52]. In grafted tomatoes, overexpression of *NCED*-mediated ABA signaling in rootstocks can improve salt tolerance [53]. In grafted pepper, H_2_O_2_ signaling in rootstocks promoted the content of phenols and proline in scions, and ABA signaling promoted the K^+^/Na^+^ balance in scions, thus improving salt tolerance [54]. Our previous study revealed that H_2_O_2_ (*CmoRBOHD1*-mediated) and ABA synthesized by pumpkin rootstocks could be transported to the scion of cucumbers, promoting stomatal closure in leaves to resist salt stress [42, 43].

In this study it was found that *CmoNCED6* is an important gene in pumpkin rootstocks for ABA production, and it is in the same branch of the phylogenetic tree as *AtNCED3* in *A. thaliana* (Fig. 1a; Supplementary Data Fig. S1). *NCED3* in *A. thaliana* and homologous *NCED* genes in other species are also key genes for ABA synthesis [55, 56], suggesting that the function of *CmoNCED6* is conserved in pumpkins. In pumpkin rootstocks, knockout or overexpression of *CmoNCED6* can affect the salt tolerance of grafted cucumbers by regulating K^+^/Na^+^ homeostasis and stomatal closure (Fig. 4), which aligns with the findings of a recent study [57]. However, in the study from Niu and co-workers, *CmoNCED6* was the gene that responds to salt stress in pumpkin leaf veins, and the expression pattern of *CmoNCED6* in roots has not been determined [57]. At 3 h of salt treatment, *CmoNCED6* knockout in roots significantly reduced the content of ABA in rootstocks and scion leaves (Fig. 4g), while overexpression showed the opposite effect, suggesting that pumpkin rootstock could generate ABA and transport it to scions under salt stress, which is consistent with the findings of our previous study [43]. At the same time, it was found that root-derived ABA signals could induce the expression of ABA synthesis genes in cucumber scion leaves under salt stress, suggesting that some ABA signals in scion leaves came from pumpkin rootstocks and some were induced by root ABA signals and synthesized in scion leaves. It was found that CmoNAC1 is a key transcription factor that regulates H_2_O_2_ and ABA signaling in grafted cucumbers under salt stress by binding to the promoters of *CmoRBOHD1* and *CmoNCED6* (Fig. 5), which is similar to the results in soybeans [32], apples [31], sweet potatoes [51], and *Arabidopsis* [37]. However, the key genes regulated by pumpkin CmoNAC1 are different from those in these species. This may be due to the differences in the promoters of key genes involved in H_2_O_2_ and ABA synthesis in different species. Y1H, LUC, and EMSA were used in this study to further verify the binding of CmoNAC1 to the promoters of *CmoRBOHD1* and *CmoNCED6*, which was more in-depth than previous studies [37]. The binding site of CmoNAC1 to the promoter of *CmoRBOHD1*/*CmoNCED6* is CGT[G/A], similar to that found in sweet potatoes but different from soybeans and apples. This diversity in binding sites among NAC transcription factors in different species suggests variations in their regulatory mechanisms. Moreover, this study found that NAC transcription factors can promote the production of H_2_O_2_ signals at the early stage of salt stress. However, other studies have shown that NAC transcription factors can activate antioxidant enzymes to maintain ROS homeostasis and help plants resist salt stress [34]. This dual role of NAC transcription factors allows them to act as early stress response triggers by inducing H_2_O_2_ production and later as regulators of antioxidant enzyme activity to counteract excessive ROS accumulation during prolonged salt stress. In addition, in other studies using grafted vegetables [42, 43, 52–54], the key transcription factors that regulate H_2_O_2_ and ABA signaling under salt stress have not been reported. This study provides deeper insights into the key transcription factors.

### CmoNAC1 improves the K^+^/Na^+^ ratio of grafted cucumbers under salt stress by binding to the promoters of *CmoAKT1;2* and *CmoHKT1;1*

Maintaining an optimal K^+^/Na^+^ balance is crucial for plants to withstand salt stress, achieved through decreasing Na^+^ uptake and increasing K^+^ absorption [2]. NAC transcription factors have emerged as key regulators in enhancing the K^+/^Na^+^ ratio under salt stress. For instance, overexpressing *VuNAC1/2* in cowpeas significantly boosts the K^+^/Na^+^ ratio in shoots and roots, thereby enhancing salt tolerance [35]. In soybeans, *GmNAC06* can maintain ion homeostasis against salt stress by regulating the K^+^/Na^+^ ratio in hairy roots [33]. In tobacco, *NtNAC053* can increase the K^+^/Na^+^ ratio to resist salt stress, and the promoter analysis revealed that there were NAC-binding sites on the K^+^ transporters *NtKAT2* and Na^+^ transporters *NHX1* and *SOS1* promoter [34]. Improving the K^+^/Na^+^ ratio represents a vital strategy to enhance salt tolerance in grafted vegetable crops. In grafted cucumbers, pumpkin rootstocks upregulate *CmoHKT1;1* expression to facilitate Na^+^ transport [58], while *CmoHAK5* in pumpkin rootstocks and *CsHAK5;3* in cucumber scion leaves to increase K^+^ absorption [20, 46].

In this study it was found that the knockout and overexpression of *CmoNAC1* in rootstocks significantly affected the K^+^/Na^+^ ratio of grafted cucumbers under salt stress (Fig. 2h and j), which was similar to results in cowpeas [35], soybeans [33], and tobacco [34], indicating that in different species NAC transcription factors can affect plant salt tolerance by regulating potassium and sodium homeostasis. In cowpeas [35] and soybeans [33], it is unclear which K^+^ and Na^+^ transporter genes are regulated by NAC transcription factors in response to salt stress. This study found that NAC1 affected K^+^ and Na^+^ transport by regulating the expression of *CmoAKT1;2*, *CmoHKT1;1*, and *CmoSOS1* in pumpkin rootstocks under salt stress (Fig. 6a and b). This is different from the mechanism by which tobacco *NAC1* regulates K^+^/Na^+^ under salt stress [34]. In tobacco, through promoter analysis, NtNAC053 binding regions were discovered in the promoters of K^+^ transporter NtKAT2 and Na^+^ transporter *NHX1* and *SOS1* [34]. In this study, LUC and EMSA experiments showed that CmoNAC1 could bind to the promoters of *CmoAKT1;2* and *CmoHKT1;1* to regulate K^+^ and Na^+^ transport (Fig. 6c–h). There was no NAC-binding site in the promoter of *CmoSOS1*, which might be because NAC1 induced the expression of *CmoSOS1* by activating H_2_O_2_ signaling. Previous research [46, 58] has demonstrated that grafting improves the K^+^/Na^+^ ratio in cucumbers under salt stress. However, the specific transcription factors responsible for regulating K^+^/Na^+^ transporters have remained elusive. In this study it was found that CmoNAC1 could improve salt tolerance of grafted cucumbers by regulating the expression of *CmoHKT1;1*, which was studied more deeply than in previous studies. In addition, we found that CmoNAC1 enhanced K^+^ absorption by regulating the expression of *CmoAKT1;1*, indicating that pumpkin rootstocks could also enhance K^+^ absorption by increasing the expression of *CmoAKT1*, which enriched the previous mechanism of pumpkin rootstocks to improve the K^+^/Na^+^ ratio by increasing the expression of *CmoHAK5* and *CmoHKT1;1* [46, 58].

Ultimately, this study sheds new light on the mechanisms by which grafting enhances salt tolerance in cucumber plants. The results of this study show that pumpkin CmoNAC1 is a key transcription factor positively regulating salt tolerance of grafted cucumbers. It can regulate the production of root H_2_O_2_- and ABA-mediated stomatal closure by binding to the promoters of *CmoRBOHD1* and *CmoNCED6*. CmoNAC1 can also bind to the promoters of *CmoAKT1;2* and *CmoHKT1;1* to regulate root K^+^/Na^+^ transport to improve the K^+^/Na^+^ ratio of grafted plants (Fig. 7).

## Materials and methods

### Generation of root knockout and overexpression of *CmoNAC1*/*CmoNCED6* of grafted cucumbers

Hefei Fengle Seed Co., Ltd. and Tianjin Kerun Agricultural Technology Co., Ltd. provided salt-tolerant pumpkin (*Cucurbita maxima × Cucurbita moschata*) cv. ‘Fenglejinjia’ and salt-sensitive cucumber seeds (*Cucumis sativus* L.) cv. ‘Jinchun No. 4’, respectively. *CmoNAC1*/*CmoNCED6* (*CmoCh01G014300*/*CmoCh16G004950*) overexpression and CRISPR/cas9 vectors were constructed according to Geng *et al*. [59]. *Escherichia coli* 5α was transformed using these vectors. *Agrobacterium* K599 was then transformed using pKSE403 empty plasmids, pKSE403 containing CmoNAC1/CmoNCED6 sgRNA, and pKSE403 containing the CmoNAC1/CmoNCED6 coding sequences, according to the K599 instruction manual (Weidi, Shanghai). The pumpkin plants were subsequently infected with the transformed *Agrobacterium* K599, and grafting was performed 4 days after infection. Non-DsRed roots were removed every 10 days and the red roots were retained. Before salt treatment, the DsRed roots of knockout plants were selected for Hi-TOM sequencing to determine gene editing efficiency, while DsRed roots of overexpressing plants were selected for qRT–PCR to characterize overexpression. The transformed grafted cucumber seedlings were grown to three leaves before being subjected to treatment with 75 mM NaCl. Each replicate consisted of six plants, and three replicates were performed. Supplementary Data Table S1 lists the primers that were used.

### Determination of morphological index

After 7 days of treatment with 75 mM NaCl, the morphological indices of cucumber scions grafted on *CmoNAC1*/*CmoNCED6* knockout or overexpression rootstocks were determined. The roots were scanned with an LA-S root scanner (Regent, Canada), and the total length, area, and volume of the roots were estimated using winRHIZO software. Additionally, the dry weight was measured using an electronic balance, following a method reported by Chen *et al*. [60].

### Determination of photosynthetic performance

Following a 7-day treatment with 75 mM NaCl, the photosynthetic performance of root knockout and overexpression *CmoNAC1*/*CmoNCED6* grafted cucumber was determined. The SPAD levels in the first authentic leaf were measured using a SPAD-502 chlorophyll meter. The maximal photochemical efficiency (*F*_v_/*F*_m_) of the first authentic leaf was determined using the Imaging PAM fluorescence analyzer (IMAG-MAXI, Germany). Additionally, measurements of Pn, stomatal conductance (*G*_s_), and intercellular CO_2_ concentration (*C*_i_) between 8:30 and 11:30 a.m. were performed on the first genuine leaf using an open gas exchange system (Li-6400, Li-Cor, Inc., Lincoln, NE, USA), as described by Chen *et al*. [60].

### Determination of malondialdehyde content and relative conductivity

The MDA content and relative conductivity (REC) in cucumber scions grafted on *CmoNAC1*/*CmoNCED6* knockout or overexpression rootstocks were determined after 7 days of treatment with 75 mM NaCl. MDA content and REC were determined using 0.1 g fresh leaf and root samples. The approach reported in previous studies was used to determine MDA content and REC [42, 60].

### Determination of Na^+^ and K^+^ content

The Na^+^ and K^+^ contents of cucumber scions grafted on *CmoNAC1*/*CmoNCED6* knockout or overexpression rootstocks were assessed after a 7-day treatment with 75 mM NaCl. Fresh root and leaf samples were dried at 80°C after being baked for 15 min at 105°C. The dried samples were then powdered and weighed, with 0.1 g of each sample transferred to a digestive tube. The samples were digested until they became clear, and the resulting digestive liquid was subsequently reduced to a volume of 50 ml. Measurements were conducted with an atomic absorption spectrophotometer (Varian Spectra AA220, USA) after appropriate dilution to achieve the suitable concentration [59].

### Determination of H_2_O_2_ and ABA content

After 3 h of NaCl treatment, the H_2_O_2_ content was determined in cucumber scions grafted on *CmoNAC1* knockout or overexpression rootstocks, and the ABA content was determined in cucumber scions grafted on *CmoNAC1*/*CmoNCED6* knockout or overexpression rootstocks. We used the hydrogen peroxide kit provided by Nanjing Jiancheng Institute of Biological Engineering, to determine the H_2_O_2_ concentration [60]. ABA content was estimated by Megi Bio (Shanghai). Samples of 50 mg were accurately weighed and 1 ml 50% acetonitrile–water solution was added; the preparation was ground at 4°C for 6 min, ultrasonicated at low temperature for 30 min (5°C, 40 kHz), stood at 4°C for 30 min, and centrifuged at 4°C for 15 min at 14 000 rcf, and 500 μl was removed into an HLB column for purification. The eluent was collected and pumped into a 2-ml centrifuge tube with nitrogen until dry, then 100 μl 50% methanol aqueous solution was placed in a vortex­mixer, ultrasonicated at low temperature for 5 min (5°C, 40 kHz), and centrifuged at 14 000 rcf at 4°C for 15 min, and 40 μl supernatant was taken into the sample vial. Finally, the ABA content in the samples was quantitatively determined by LC–ESI–MS/MS (UHPLC-Qtrap) by Megi Bio, Shanghai.

### Transcriptome assay and quantitative real-time PCR analysis

The transcriptome data of pumpkin root tips were obtained from https://www.ncbi.nlm.nih.gov/bioproject/PRJNA437579 [46], and the transcriptome data of root of grafted cucumber and self-grafted pumpkin were obtained from http://www.ncbi.nlm.nih.gov/bioproject/952931 [20]. For the root knockout and overexpression *CmoNAC1* grafted cucumber samples, leaf and root samples were collected after a 3-h treatment with 75 mM NaCl. The collected samples were sent to Megi Bio, Shanghai, for RNA sequencing. In the differential expression analysis, the cucumber (‘Chinese Long’) genome V3 (http://cucurbitgenomics.org/) was employed, and the software DESeq2 was used to compute and identify differentially expressed genes [61]. Gene expression was assessed using TPM, and the criteria for differential gene screening were set as log_2_(fold change value) > 1.0, *P*-value <.05 [62]. The RNA-seq data have been submitted to NCBI and can be accessed under the accession number PRJNA973836 (https://www.ncbi.nlm.nih.gov/bioproject/973836). qRT–PCR analysis was performed using the method described in a previous paper [60]; Supplementary Data Table S1 provides the primers used.

### Identification of NCED/RBOH proteins in pumpkin and cucumber

The protein sequences of cucumber, pumpkin, and *Arabidopsis* were obtained from publicly available databases (http://cucurbitgenomics.org/;http://www.arabidopsis.org/). To compare the protein sequences of cucumber and pumpkin with the *A. thaliana* NCED/RBOH protein sequence, BLASTP was used. The *Arabidopsis* NCED/RBOH protein sequence was used as the query sequence in the BLASTP search. Protein sequences with >50% similarity and a threshold of <10^−10^ were screened. To obtain hidden Markov models for NCED (PF03055) and RBOH (PF08414, PF01794, PF08022, and PF08030), the Pfam database was utilized. The hidden Markov models were downloaded from the Pfam database, which can be accessed at https://www.ebi.ac.uk/interpro/.

### Analysis of *CmoRBOHD1*/*CmoNCED6* promoters and K^+^/Na^+^ transporter genes

*CmoRBOHD1*/*CmoNCED6* and K^+^/Na^+^ transporter gene promoters 2000 bp before the ATG were downloaded from a publicly available database (http://cucurbitgenomics.org/), and their promoters were submitted to the Plant Transcription Factor Database, which can be accessed at http://planttfdb.gao-lab.org/prediction.php, and *Arabidopsis* transcription factors and binding sites that bind to it were obtained. Using BLASTP, the protein sequences of cucumber and pumpkin were compared with the transcription factor sequence from *A. thaliana* as the query sequence. As transcription factors that bind to the promoters of *CmoRBOHD1*/*CmoNCED6* and K^+^/Na^+^ transporter genes, protein sequences with a similarity >50% and a threshold of <10^−10^ were screened. Supplementary Data Table S2 provides the K^+^/Na^+^ transporter gene accession numbers.

### Yeast one-hybrid assay

In the Y1H analysis [63], pGADKT7 and pHis2 were used as expression vectors and CmoNAC1 primers were used for PCR cloning of the coding sequence. The vector pGADKT7 was linearized by the BamHI restriction endonuclease and the cloned fragment was ligated into the vector using the BamHI restriction site. Then, the NACbs on the promoters of *CmoRBOHD1*, *CmoNCED6*/*CmoAKT1;2*, and *CmoHKT1;1* were synthesized. Yeast was co-transformed with pGADKT7 and pHis2-NACbs. Yeast co-transformed with pGADKT7 empty vector and pHis2-NACbs were used as a negative control to determine the self-activation concentration of 3-AT, while the positive control was yeast co-transformed with pGADKT7–53 and pHis2–53. Then, the monoclonal strains grown on solid SD/−Leu/−Trp were inoculated into SD/−Leu/−Trp liquid medium with sterilized toothpicks and cultured overnight in a shaker at 28°C at 200 rpm/min until OD600 = 0.5–1.0. After culture the OD600 value was measured. The OD600 value of the yeast solution was uniformly adjusted to 0.1, and the yeast solution was diluted with sterilized ddH_2_O. Then, the above three kinds of diluted yeast solution were dropped on the solid medium of SD/−Trp−Leu−His+0/40/80 mM 3-AT, and then dried on the plate. The plates were placed in an inverted 28°C incubator for 3–5 days and the results were observed.

### Luciferase assay

*Nicotiana benthamiana* leaves were used in the dual-LUC activity experiment. Primers were designed to amplify the full length of *CmoNAC1*, and the amplified fragment was inserted between the BamHI and XhoI restriction sites of the pGreenII62-SK vector. The NACbs of the promoters of *CmoRBOHD1*, *CmoNCED6*, *CmoAKT1;2*, and *CmoHKT1;1* were also inserted into the pGreenII0800-LUC vector using KpnI and SmaI sites. The constructed plasmids were transferred into *Agrobacterium* GV3101 and transformed into *N. benthamiana*. Dual-LUC reporter gene test kits from Beyotime (Shanghai, China) and TecaninfiniteM200Pro (Tecan) were used to determine the LUC/REN ratio. NightSHADELB985 (Berthold, Germany) was used to observe the fluorescence activity.

### Electrophoretic mobility shift assay

The EMSA was performed as described previously [64]. To express GST-tagged protein, the *CmoNAC1* coding sequence was incorporated into pGEX-4 T-2 plasmids. Isopropyl β-d-1-thiogalactopyranoside (0.2 mM) was used to induce the expression of recombinant GST-CmoNAC1 protein in BL21 *E. coli* Rosetta (DE3) for 16 h at 18°C under constant shaking. The recombinant protein was purified using GST-Sefinose™ Resin from Sangon, Shanghai, China. The purified proteins were quantified using a bicinchoninic acid protein assay kit (Beyotime, Shanghai, China). 6-FAM-labeled probes containing NACbs of the promoters of *CmoRBOHD1*, *CmoNCED6*, *CmoAKT1;2*, and *CmoHKT1;1* were synthesized by Sangon Biotech (Shanghai, China). The purified protein and labeled probes were incubated together at 4°C for 30 min to allow binding. The samples were run on the gel [2 ml TBE buffer (5×), 4 ml 30% polyacrylamide, 1 ml 50% glycerin, 100 μl 10% ammonium persulfate, 20 μl TEMED, 12.88 ml ddH_2_O] at 4°C and 100 V for 1 h in 0.59 Tris–borate EDTA buffer. After electrophoresis, the electrophoresis tank was quickly opened and the rubber plate was taken out. After the surface was rinsed with deionized water, the rubber plate was quickly transferred to the dark environment, and a multi-color fluorescence and chemiluminescence imager (FM 1038, USA, Bio-Techne corporation) was used to scan and take photographs.

### Analysis of images and data

Statistical analysis and graphical display of the data were performed using the RStudio 4.03 program. To determine significant differences, ‘Duncan’s new multiple range test was used.

## Supplementary Material

Web_Material_uhad157

## Data Availability

The authors declare that all the data necessary to support the study’s conclusions are included in the paper and the supplementary materials, or can be obtained upon request from the corresponding author.
