# Supplementary material for: CmoNAC1 in pumpkin rootstocks improves salt tolerance of grafted cucumbers by binding to the promoters of CmoRBOHD1, CmoNCED6, CmoAKT1;2 and CmoHKT1;1 to regulate H2O2, ABA signaling and K+/Na+ homeostasis
Source: Hortic Res. 2023 Jul 25;10(9):uhad157. doi: 10.1093/hr/uhad157 (PMC10500151; doi:10.1093/hr/uhad157)
Supplement: Web_Material_uhad157 [file web_material_uhad157.zip › Supplementary material.pptx]

## Slide 1
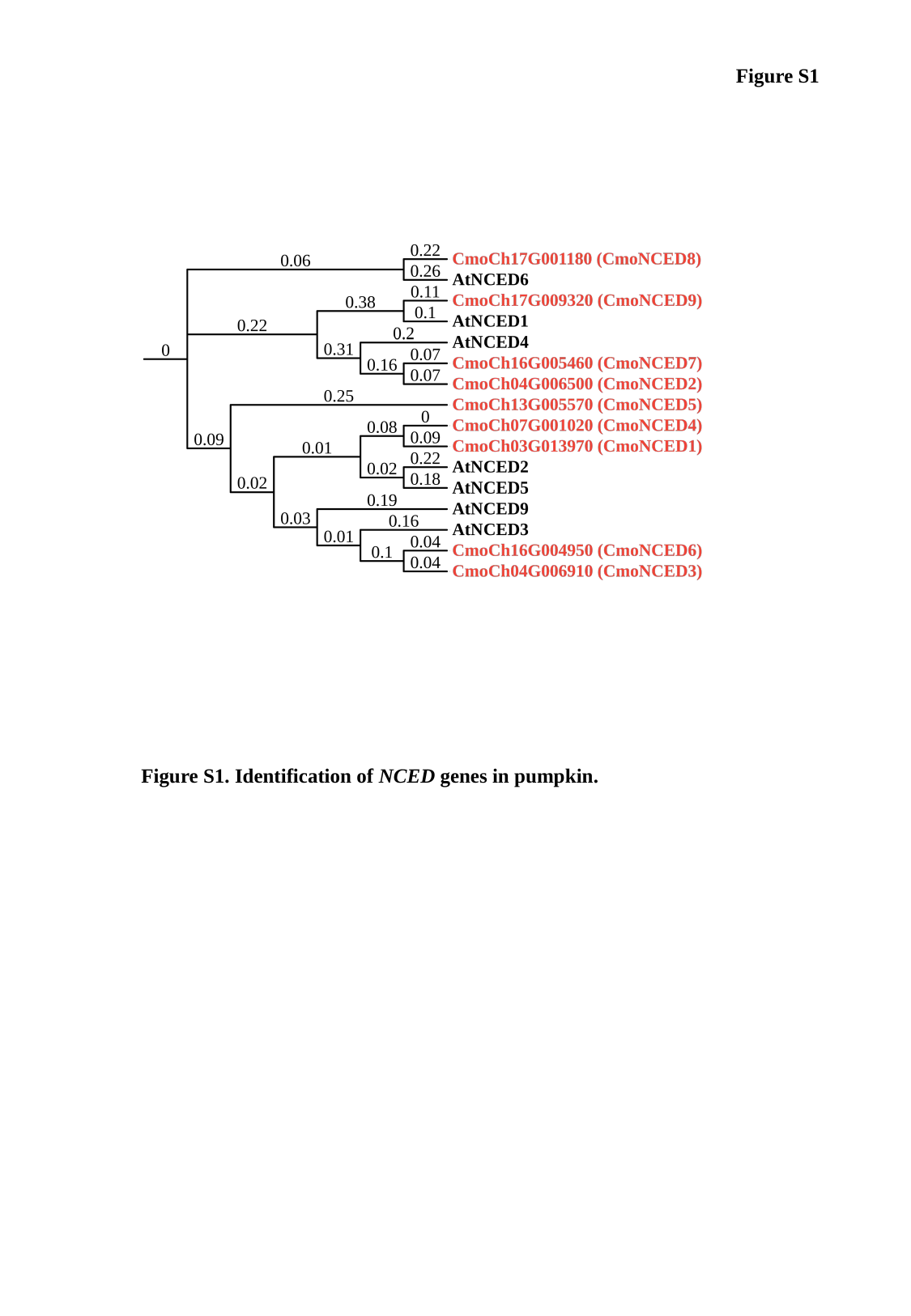

Figure S1
Figure S1. Identification of NCED genes in pumpkin.

## Slide 2
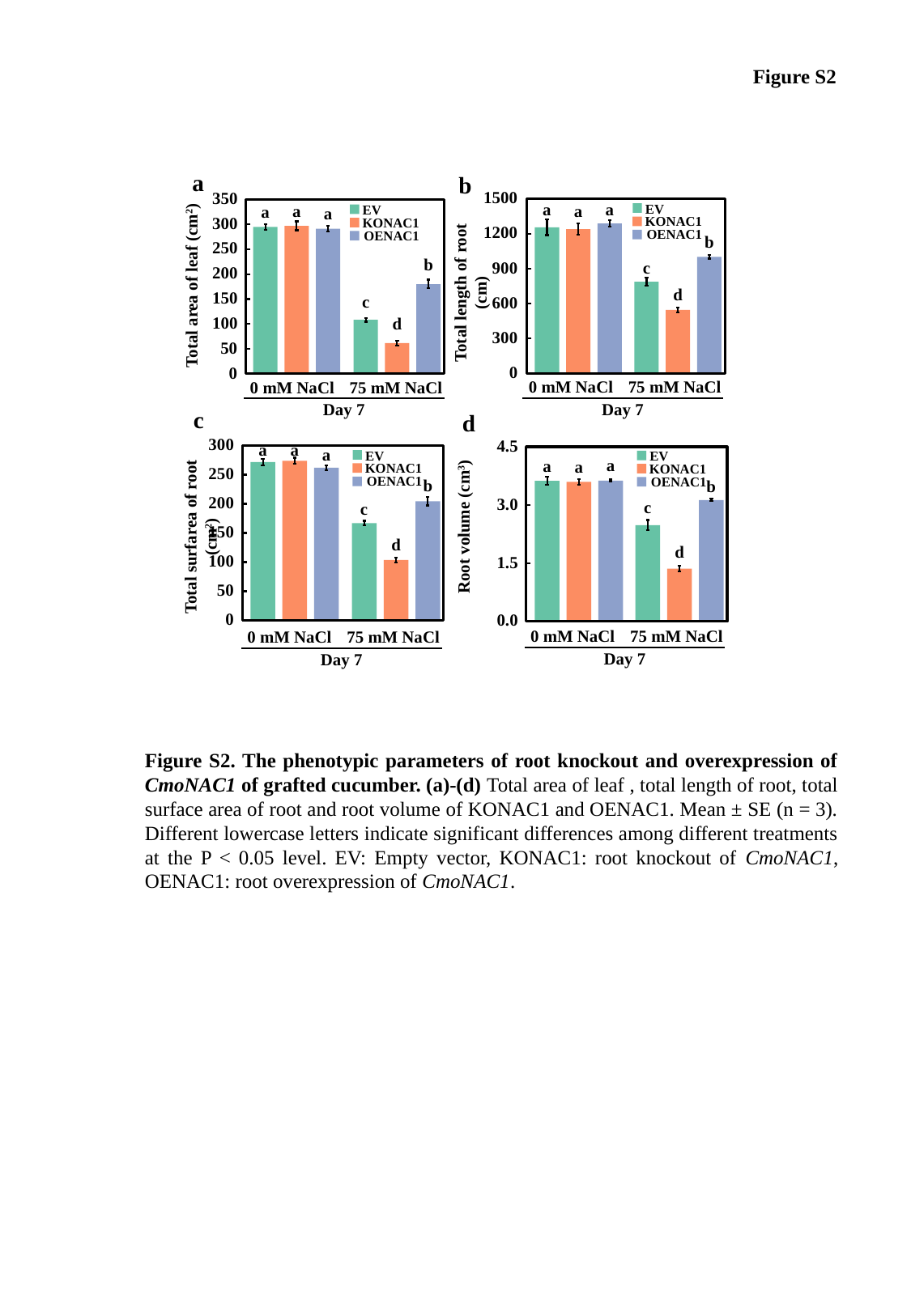

Figure S2
a
b
Total area of leaf (cm2)
1500
a
a
a
1200
b
900
c
d
600
300
0
350
a
a
a
300
250
b
200
150
c
100
d
50
0
Total length of root (cm)
EV
KONAC1
 OENAC1
EV
KONAC1
 OENAC1
0 mM NaCl
75 mM NaCl
Day 7
0 mM NaCl
75 mM NaCl
Day 7
c
d
Total surfarea of root (cm2)
300
a
a
a
250
b
200
c
150
d
100
50
0
4.5
a
a
a
b
3.0
c
d
1.5
0.0
Root volume (cm3)
EV
KONAC1
 OENAC1
EV
KONAC1
 OENAC1
0 mM NaCl
75 mM NaCl
Day 7
0 mM NaCl
75 mM NaCl
Day 7
Figure S2. The phenotypic parameters of root knockout and overexpression of CmoNAC1 of grafted cucumber. (a)-(d) Total area of leaf , total length of root, total surface area of root and root volume of KONAC1 and OENAC1. Mean ± SE (n = 3). Different lowercase letters indicate significant differences among different treatments at the P < 0.05 level. EV: Empty vector, KONAC1: root knockout of CmoNAC1, OENAC1: root overexpression of CmoNAC1.

## Slide 3
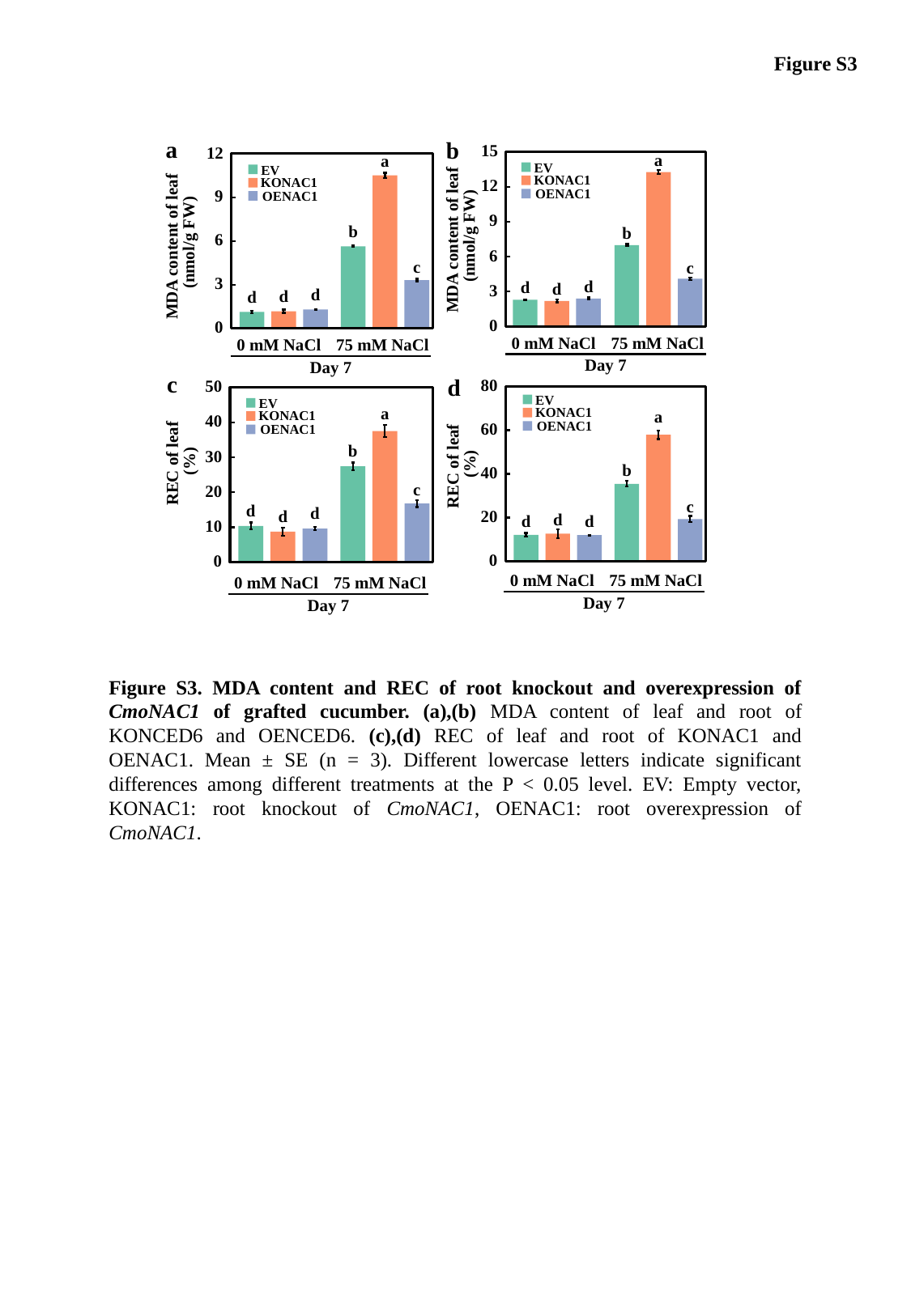

Figure S3
a
b
15
a
12
9
b
6
c
d
d
d
3
0
12
a
9
b
6
c
3
d
d
d
0
EV
KONAC1
 OENAC1
EV
KONAC1
 OENAC1
MDA content of leaf
 (nmol/g FW)
MDA content of leaf
 (nmol/g FW)
0 mM NaCl
75 mM NaCl
Day 7
0 mM NaCl
75 mM NaCl
Day 7
c
d
80
50
EV
KONAC1
 OENAC1
EV
KONAC1
 OENAC1
a
a
40
60
REC of leaf
 (%)
b
REC of leaf
 (%)
30
b
40
c
20
c
d
d
d
20
d
d
d
10
0
0
0 mM NaCl
75 mM NaCl
Day 7
0 mM NaCl
75 mM NaCl
Day 7
Figure S3. MDA content and REC of root knockout and overexpression of CmoNAC1 of grafted cucumber. (a),(b) MDA content of leaf and root of KONCED6 and OENCED6. (c),(d) REC of leaf and root of KONAC1 and OENAC1. Mean ± SE (n = 3). Different lowercase letters indicate significant differences among different treatments at the P < 0.05 level. EV: Empty vector, KONAC1: root knockout of CmoNAC1, OENAC1: root overexpression of CmoNAC1.

## Slide 4
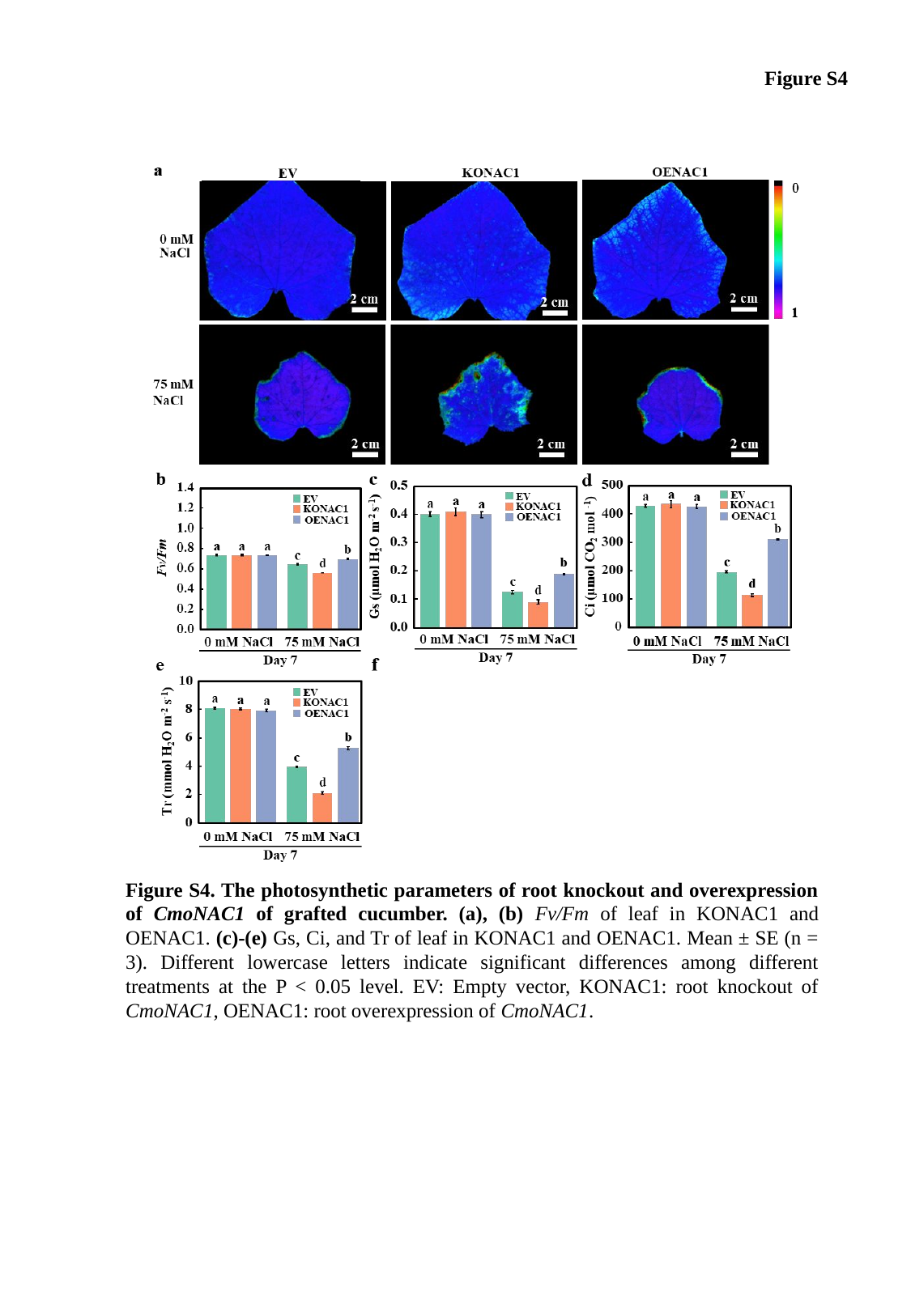

Figure S4
Figure S4. The photosynthetic parameters of root knockout and overexpression of CmoNAC1 of grafted cucumber. (a), (b) Fv/Fm of leaf in KONAC1 and OENAC1. (c)-(e) Gs, Ci, and Tr of leaf in KONAC1 and OENAC1. Mean ± SE (n = 3). Different lowercase letters indicate significant differences among different treatments at the P < 0.05 level. EV: Empty vector, KONAC1: root knockout of CmoNAC1, OENAC1: root overexpression of CmoNAC1.

## Slide 5
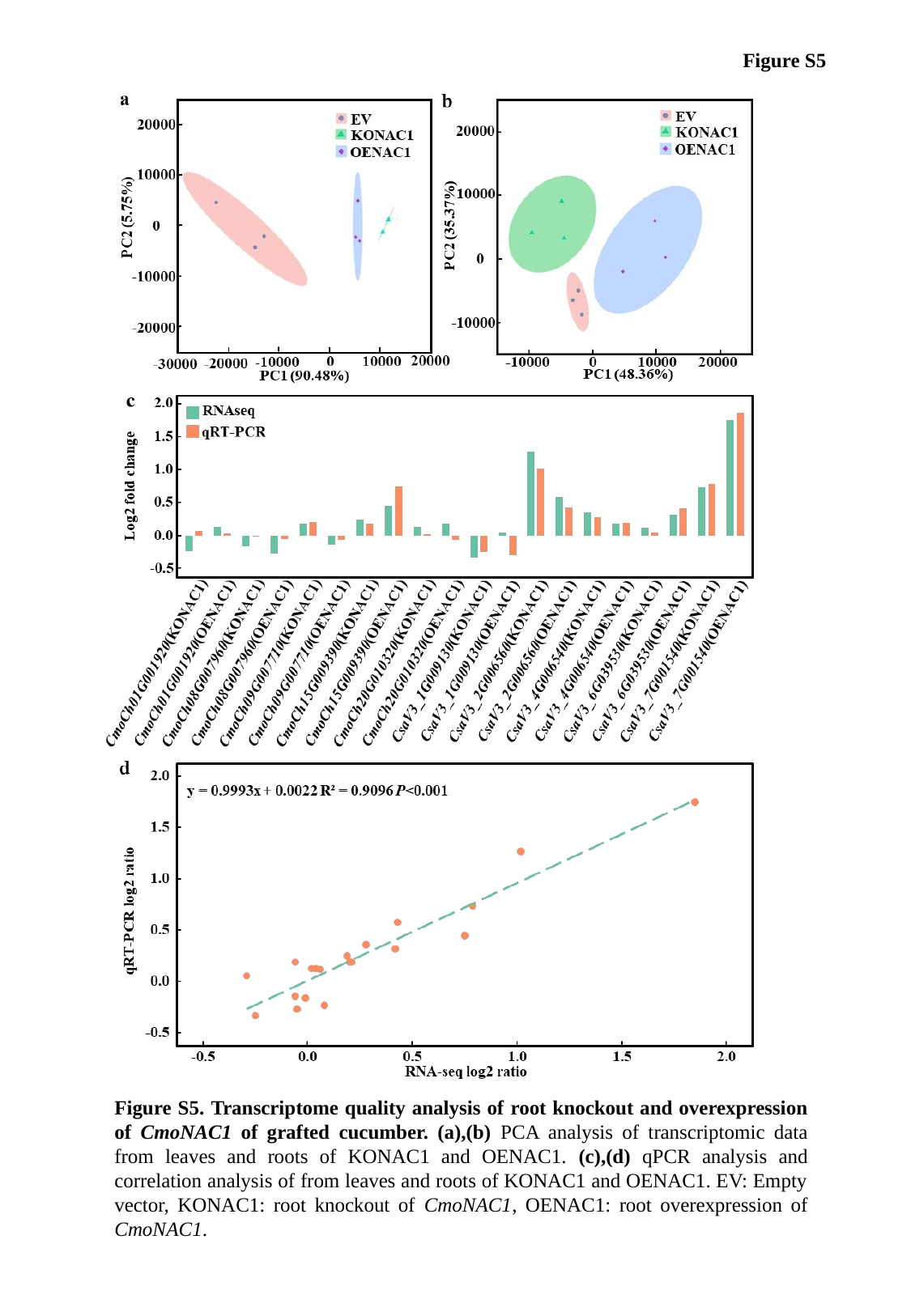

Figure S5
Figure S5. Transcriptome quality analysis of root knockout and overexpression of CmoNAC1 of grafted cucumber. (a),(b) PCA analysis of transcriptomic data from leaves and roots of KONAC1 and OENAC1. (c),(d) qPCR analysis and correlation analysis of from leaves and roots of KONAC1 and OENAC1. EV: Empty vector, KONAC1: root knockout of CmoNAC1, OENAC1: root overexpression of CmoNAC1.

## Slide 6
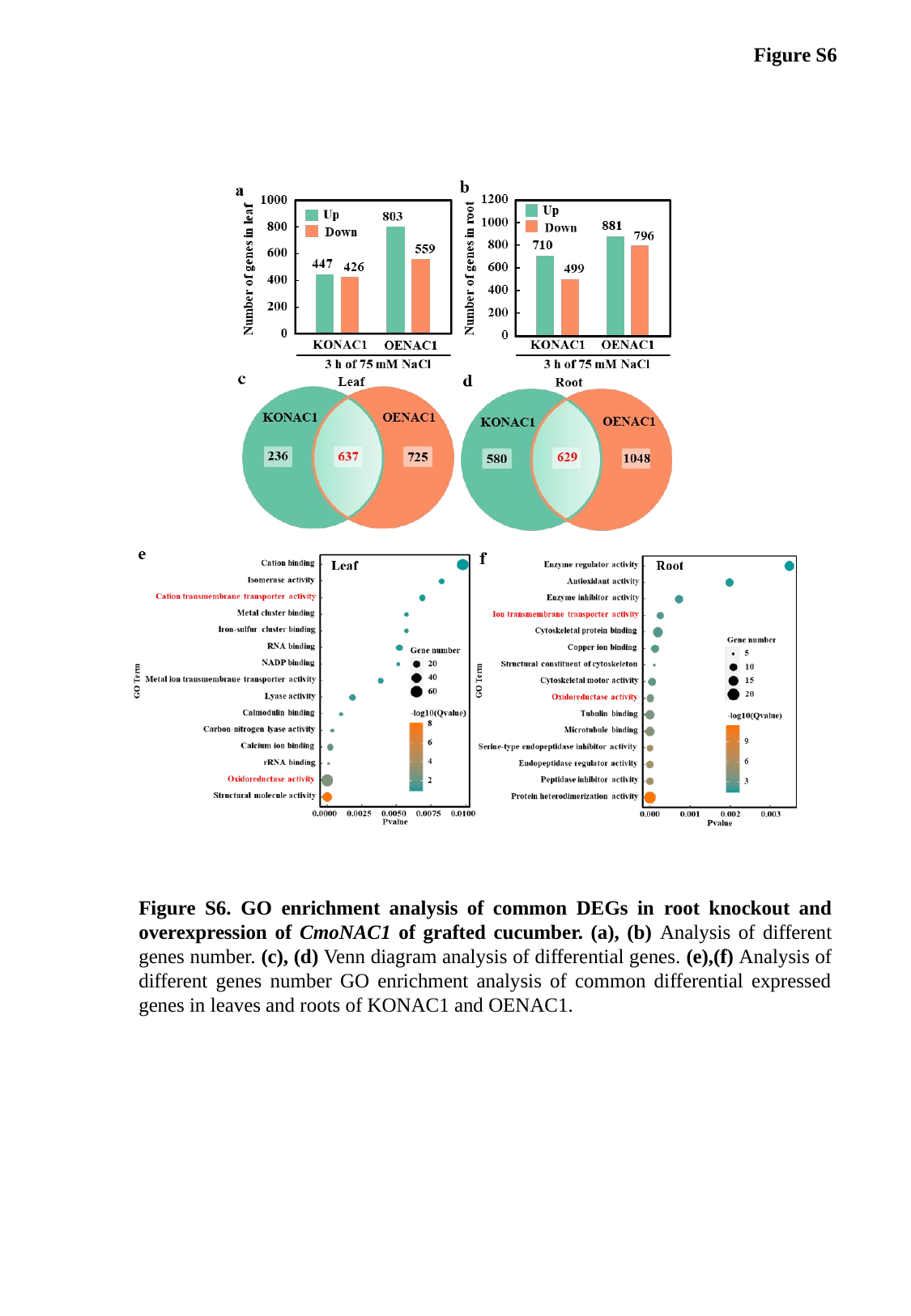

Figure S6
Figure S6. GO enrichment analysis of common DEGs in root knockout and overexpression of CmoNAC1 of grafted cucumber. (a), (b) Analysis of different genes number. (c), (d) Venn diagram analysis of differential genes. (e),(f) Analysis of different genes number GO enrichment analysis of common differential expressed genes in leaves and roots of KONAC1 and OENAC1.

## Slide 7
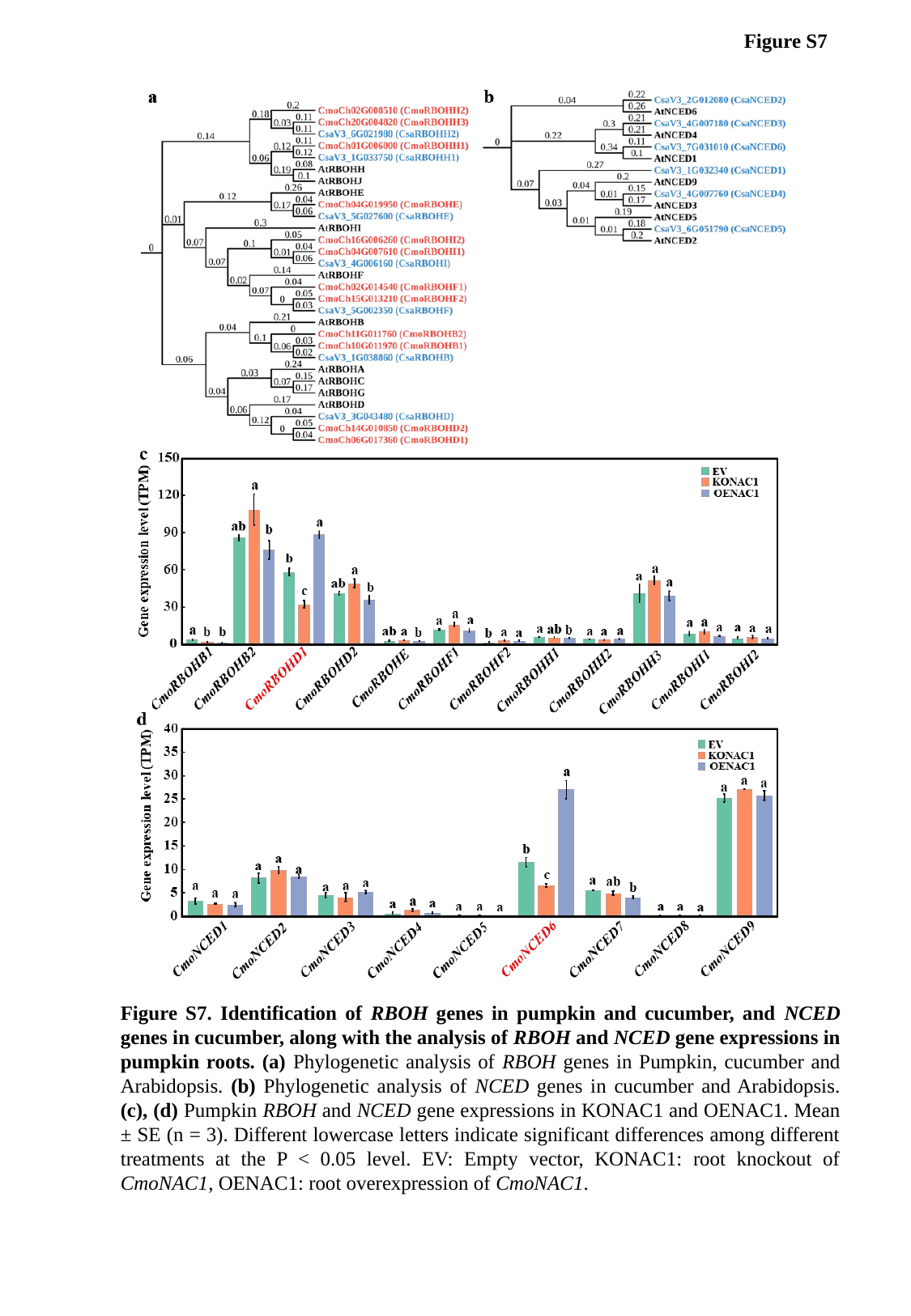

Figure S7
Figure S7. Identification of RBOH genes in pumpkin and cucumber, and NCED genes in cucumber, along with the analysis of RBOH and NCED gene expressions in pumpkin roots. (a) Phylogenetic analysis of RBOH genes in Pumpkin, cucumber and Arabidopsis. (b) Phylogenetic analysis of NCED genes in cucumber and Arabidopsis. (c), (d) Pumpkin RBOH and NCED gene expressions in KONAC1 and OENAC1. Mean ± SE (n = 3). Different lowercase letters indicate significant differences among different treatments at the P < 0.05 level. EV: Empty vector, KONAC1: root knockout of CmoNAC1, OENAC1: root overexpression of CmoNAC1.

## Slide 8
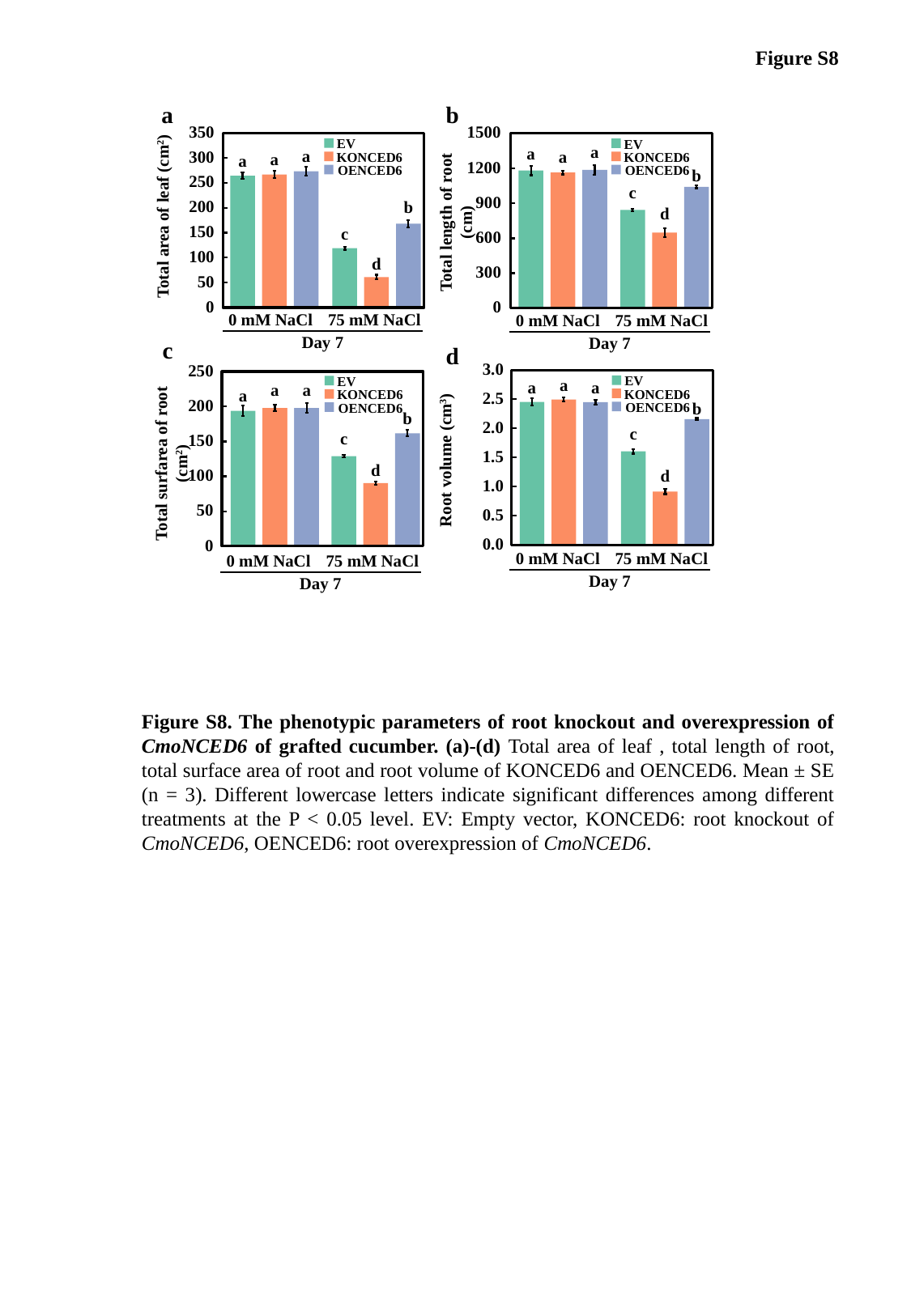

Figure S8
b
a
Total area of leaf (cm2)
Total length of root (cm)
350
1500
EV
KONCED6
OENCED6
EV
KONCED6
OENCED6
a
a
300
a
a
a
a
1200
b
250
c
900
200
b
d
150
c
600
100
d
300
50
0
0
0 mM NaCl
75 mM NaCl
Day 7
0 mM NaCl
75 mM NaCl
Day 7
c
d
Total surfarea of root (cm2)
3.0
250
EV
KONCED6
OENCED6
EV
KONCED6
OENCED6
Root volume (cm3)
a
a
a
a
a
a
2.5
200
b
b
2.0
c
c
150
1.5
d
100
d
1.0
50
0.5
0.0
0
0 mM NaCl
75 mM NaCl
Day 7
0 mM NaCl
75 mM NaCl
Day 7
Figure S8. The phenotypic parameters of root knockout and overexpression of CmoNCED6 of grafted cucumber. (a)-(d) Total area of leaf , total length of root, total surface area of root and root volume of KONCED6 and OENCED6. Mean ± SE (n = 3). Different lowercase letters indicate significant differences among different treatments at the P < 0.05 level. EV: Empty vector, KONCED6: root knockout of CmoNCED6, OENCED6: root overexpression of CmoNCED6.

## Slide 9
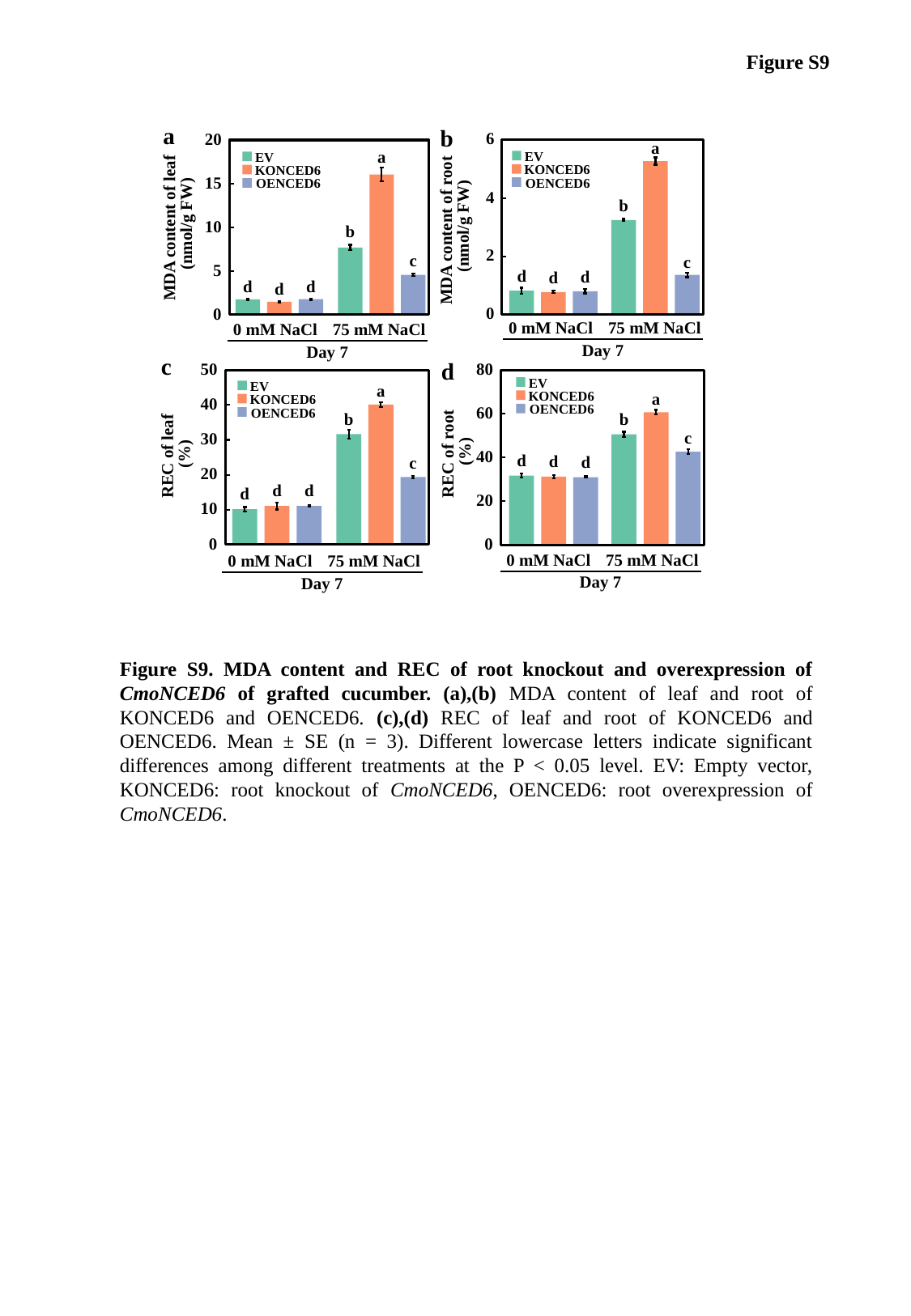

Figure S9
a
b
6
a
4
b
2
c
d
d
d
0
20
EV
KONCED6
OENCED6
EV
KONCED6
OENCED6
a
15
MDA content of leaf
 (nmol/g FW)
MDA content of root
 (nmol/g FW)
10
b
c
5
d
d
d
0
0 mM NaCl
75 mM NaCl
Day 7
0 mM NaCl
75 mM NaCl
Day 7
c
d
50
a
40
b
30
c
20
d
d
d
10
0
80
a
60
b
c
40
d
d
d
20
0
EV
KONCED6
OENCED6
EV
KONCED6
OENCED6
REC of root
 (%)
REC of leaf
 (%)
0 mM NaCl
75 mM NaCl
Day 7
0 mM NaCl
75 mM NaCl
Day 7
Figure S9. MDA content and REC of root knockout and overexpression of CmoNCED6 of grafted cucumber. (a),(b) MDA content of leaf and root of KONCED6 and OENCED6. (c),(d) REC of leaf and root of KONCED6 and OENCED6. Mean ± SE (n = 3). Different lowercase letters indicate significant differences among different treatments at the P < 0.05 level. EV: Empty vector, KONCED6: root knockout of CmoNCED6, OENCED6: root overexpression of CmoNCED6.

## Slide 10
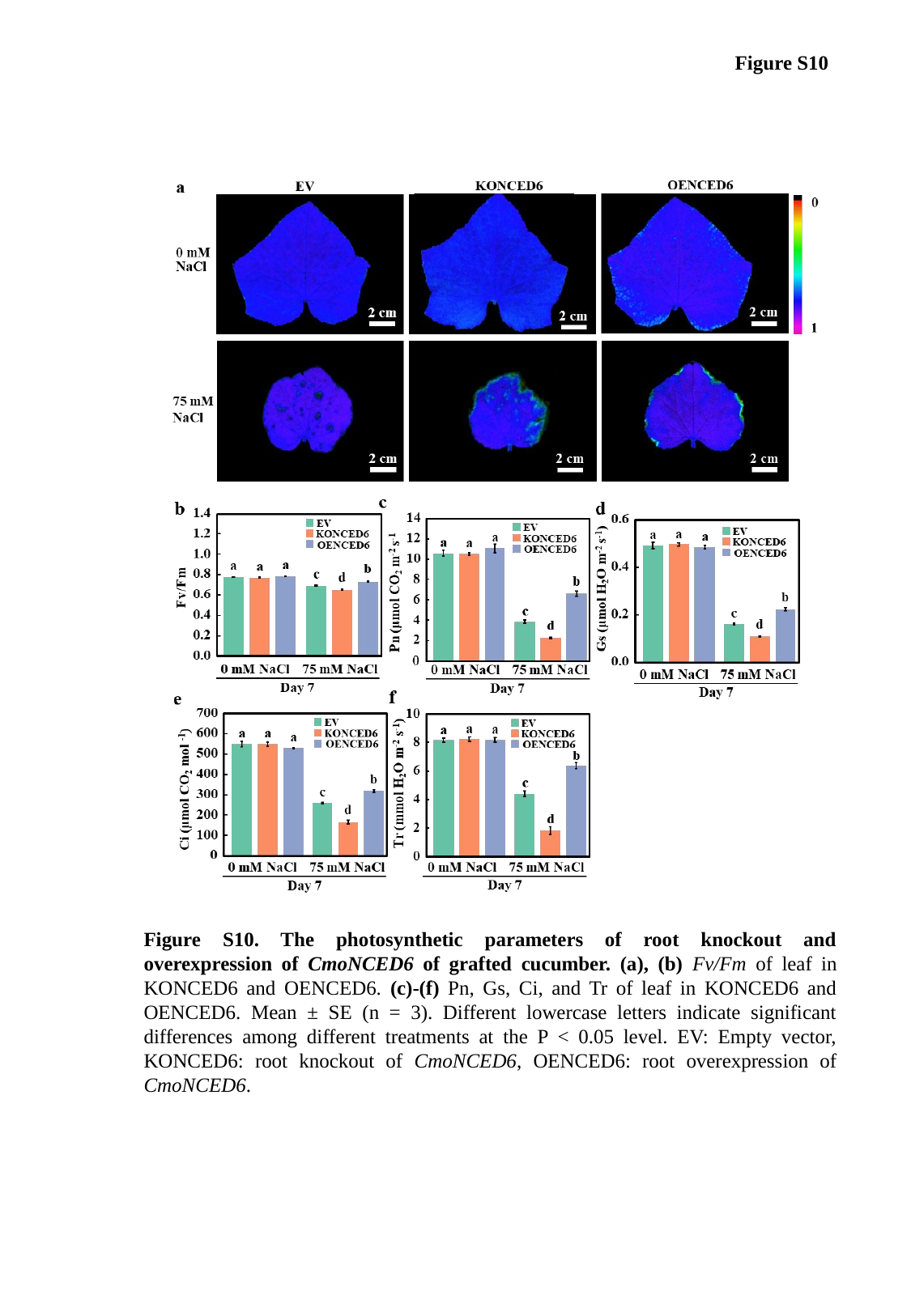

Figure S10
Figure S10. The photosynthetic parameters of root knockout and overexpression of CmoNCED6 of grafted cucumber. (a), (b) Fv/Fm of leaf in KONCED6 and OENCED6. (c)-(f) Pn, Gs, Ci, and Tr of leaf in KONCED6 and OENCED6. Mean ± SE (n = 3). Different lowercase letters indicate significant differences among different treatments at the P < 0.05 level. EV: Empty vector, KONCED6: root knockout of CmoNCED6, OENCED6: root overexpression of CmoNCED6.

## Slide 11
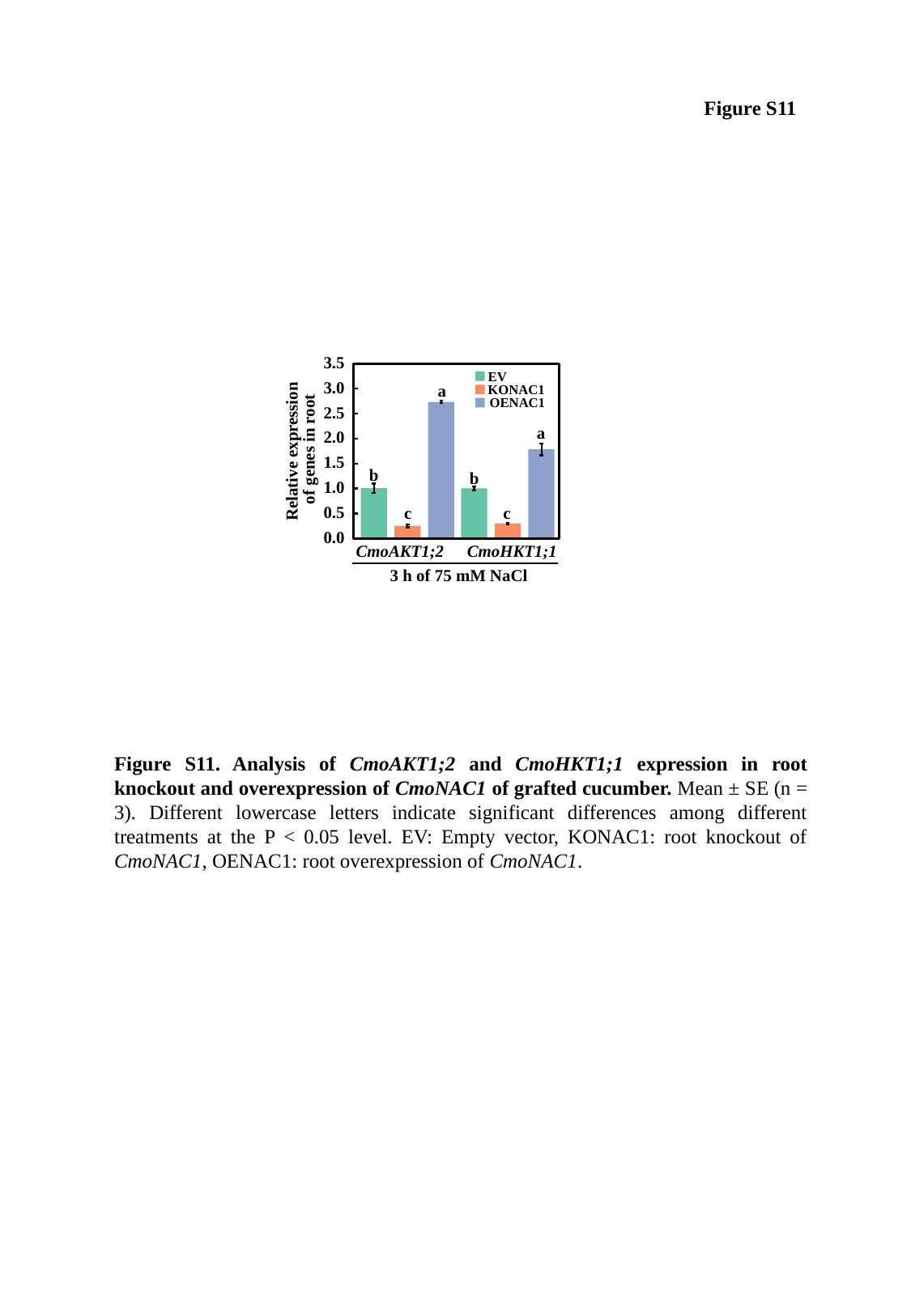

Figure S11
3.5
EV
KONAC1
 OENAC1
3.0
a
2.5
a
Relative expression
of genes in root
2.0
1.5
b
b
1.0
c
c
0.5
0.0
CmoAKT1;2
CmoHKT1;1
3 h of 75 mM NaCl
Figure S11. Analysis of CmoAKT1;2 and CmoHKT1;1 expression in root knockout and overexpression of CmoNAC1 of grafted cucumber. Mean ± SE (n = 3). Different lowercase letters indicate significant differences among different treatments at the P < 0.05 level. EV: Empty vector, KONAC1: root knockout of CmoNAC1, OENAC1: root overexpression of CmoNAC1.

## Slide 12
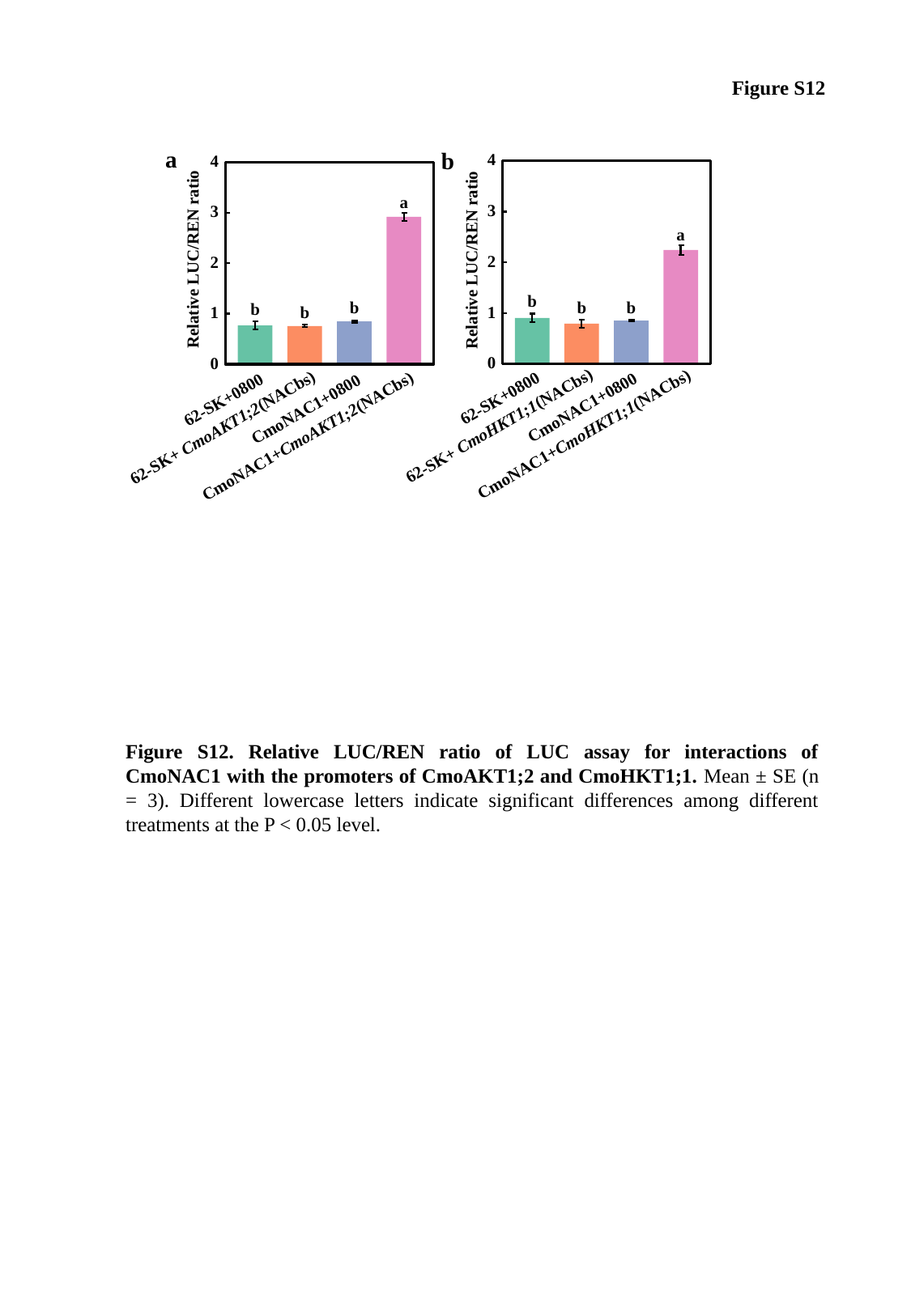

Figure S12
a
4
a
3
2
b
b
b
1
0
Relative LUC/REN ratio
62-SK+0800
CmoNAC1+0800
62-SK+ CmoAKT1;2(NACbs)
CmoNAC1+CmoAKT1;2(NACbs)
b
4
3
a
2
b
b
b
1
0
Relative LUC/REN ratio
62-SK+0800
CmoNAC1+0800
62-SK+ CmoHKT1;1(NACbs)
CmoNAC1+CmoHKT1;1(NACbs)
Figure S12. Relative LUC/REN ratio of LUC assay for interactions of CmoNAC1 with the promoters of CmoAKT1;2 and CmoHKT1;1. Mean ± SE (n = 3). Different lowercase letters indicate significant differences among different treatments at the P < 0.05 level.

## Slide 13
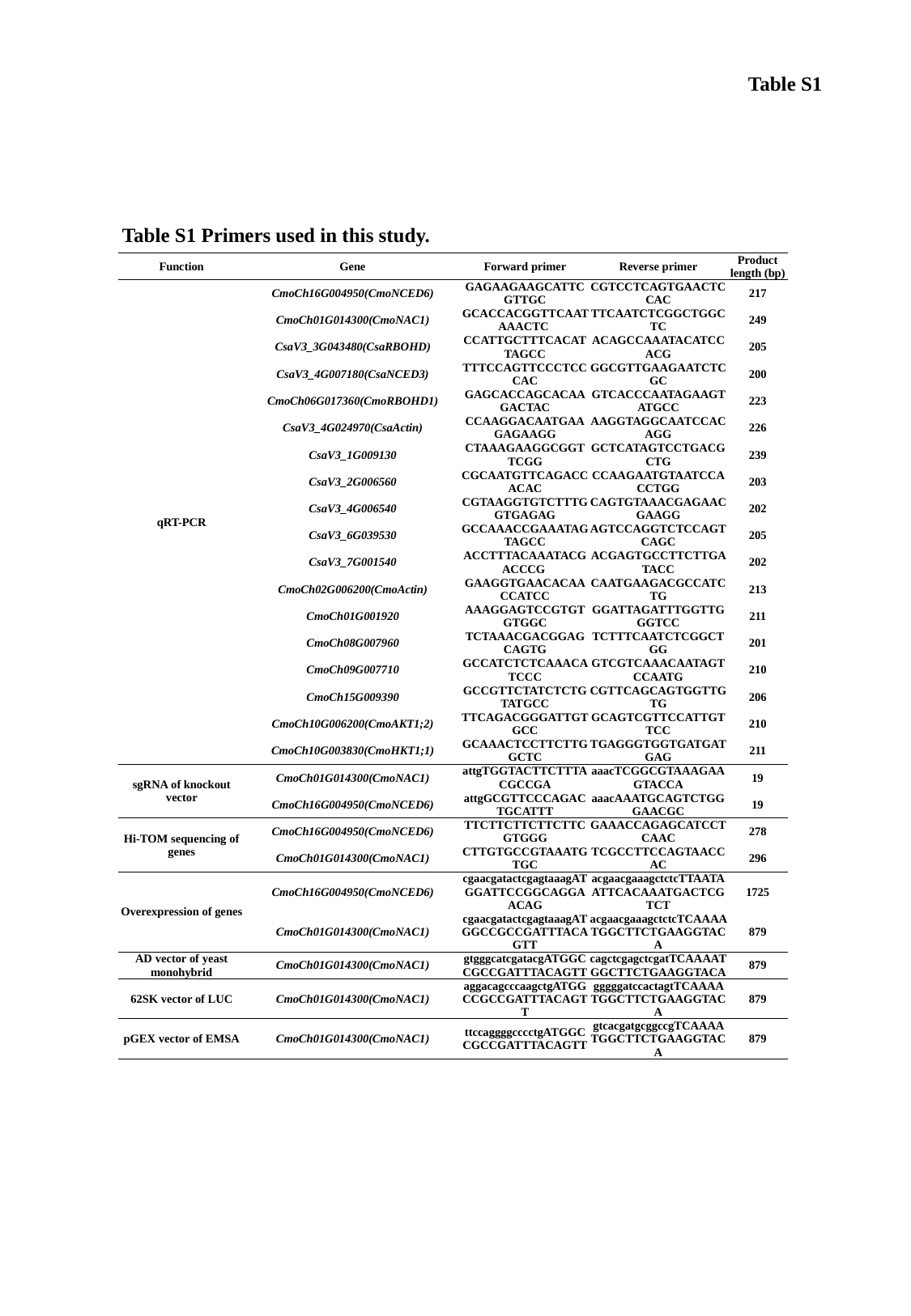

Table S1
Table S1 Primers used in this study.
| Function | Gene | Forward primer | Reverse primer | Product length (bp) |
| --- | --- | --- | --- | --- |
| qRT-PCR | CmoCh16G004950(CmoNCED6) | GAGAAGAAGCATTCGTTGC | CGTCCTCAGTGAACTCCAC | 217 |
| | CmoCh01G014300(CmoNAC1) | GCACCACGGTTCAATAAACTC | TTCAATCTCGGCTGGCTC | 249 |
| | CsaV3\_3G043480(CsaRBOHD) | CCATTGCTTTCACATTAGCC | ACAGCCAAATACATCCACG | 205 |
| | CsaV3\_4G007180(CsaNCED3) | TTTCCAGTTCCCTCCCAC | GGCGTTGAAGAATCTCGC | 200 |
| | CmoCh06G017360(CmoRBOHD1) | GAGCACCAGCACAAGACTAC | GTCACCCAATAGAAGTATGCC | 223 |
| | CsaV3\_4G024970(CsaActin) | CCAAGGACAATGAAGAGAAGG | AAGGTAGGCAATCCACAGG | 226 |
| | CsaV3\_1G009130 | CTAAAGAAGGCGGTTCGG | GCTCATAGTCCTGACGCTG | 239 |
| | CsaV3\_2G006560 | CGCAATGTTCAGACCACAC | CCAAGAATGTAATCCACCTGG | 203 |
| | CsaV3\_4G006540 | CGTAAGGTGTCTTTGGTGAGAG | CAGTGTAAACGAGAACGAAGG | 202 |
| | CsaV3\_6G039530 | GCCAAACCGAAATAGTAGCC | AGTCCAGGTCTCCAGTCAGC | 205 |
| | CsaV3\_7G001540 | ACCTTTACAAATACGACCCG | ACGAGTGCCTTCTTGATACC | 202 |
| | CmoCh02G006200(CmoActin) | GAAGGTGAACACAACCATCC | CAATGAAGACGCCATCTG | 213 |
| | CmoCh01G001920 | AAAGGAGTCCGTGTGTGGC | GGATTAGATTTGGTTGGGTCC | 211 |
| | CmoCh08G007960 | TCTAAACGACGGAGCAGTG | TCTTTCAATCTCGGCTGG | 201 |
| | CmoCh09G007710 | GCCATCTCTCAAACATCCC | GTCGTCAAACAATAGTCCAATG | 210 |
| | CmoCh15G009390 | GCCGTTCTATCTCTGTATGCC | CGTTCAGCAGTGGTTGTG | 206 |
| | CmoCh10G006200(CmoAKT1;2) | TTCAGACGGGATTGTGCC | GCAGTCGTTCCATTGTTCC | 210 |
| | CmoCh10G003830(CmoHKT1;1) | GCAAACTCCTTCTTGGCTC | TGAGGGTGGTGATGATGAG | 211 |
| sgRNA of knockout vector | CmoCh01G014300(CmoNAC1) | attgTGGTACTTCTTTACGCCGA | aaacTCGGCGTAAAGAAGTACCA | 19 |
| | CmoCh16G004950(CmoNCED6) | attgGCGTTCCCAGACTGCATTT | aaacAAATGCAGTCTGGGAACGC | 19 |
| Hi-TOM sequencing of genes | CmoCh16G004950(CmoNCED6) | TTCTTCTTCTTCTTCGTGGG | GAAACCAGAGCATCCTCAAC | 278 |
| | CmoCh01G014300(CmoNAC1) | CTTGTGCCGTAAATGTGC | TCGCCTTCCAGTAACCAC | 296 |
| Overexpression of genes | CmoCh16G004950(CmoNCED6) | cgaacgatactcgagtaaagATGGATTCCGGCAGGAACAG | acgaacgaaagctctcTTAATAATTCACAAATGACTCGTCT | 1725 |
| | CmoCh01G014300(CmoNAC1) | cgaacgatactcgagtaaagATGGCCGCCGATTTACAGTT | acgaacgaaagctctcTCAAAATGGCTTCTGAAGGTACA | 879 |
| AD vector of yeast monohybrid | CmoCh01G014300(CmoNAC1) | gtgggcatcgatacgATGGCCGCCGATTTACAGTT | cagctcgagctcgatTCAAAATGGCTTCTGAAGGTACA | 879 |
| 62SK vector of LUC | CmoCh01G014300(CmoNAC1) | aggacagcccaagctgATGGCCGCCGATTTACAGTT | gggggatccactagtTCAAAATGGCTTCTGAAGGTACA | 879 |
| pGEX vector of EMSA | CmoCh01G014300(CmoNAC1) | ttccaggggcccctgATGGCCGCCGATTTACAGTT | gtcacgatgcggccgTCAAAATGGCTTCTGAAGGTACA | 879 |

## Slide 14
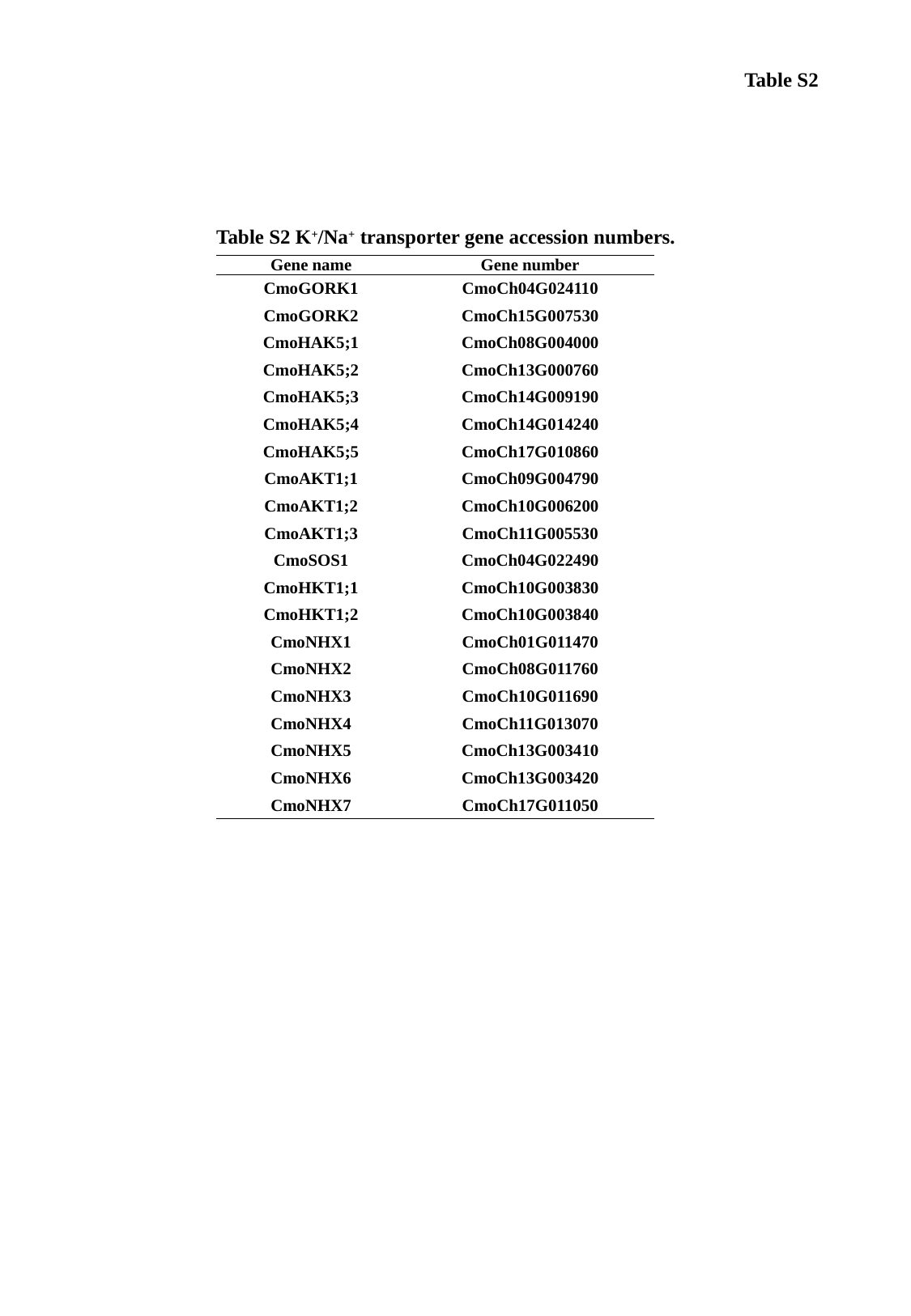

Table S2
Table S2 K+/Na+ transporter gene accession numbers.
| Gene name | Gene number |
| --- | --- |
| CmoGORK1 | CmoCh04G024110 |
| CmoGORK2 | CmoCh15G007530 |
| CmoHAK5;1 | CmoCh08G004000 |
| CmoHAK5;2 | CmoCh13G000760 |
| CmoHAK5;3 | CmoCh14G009190 |
| CmoHAK5;4 | CmoCh14G014240 |
| CmoHAK5;5 | CmoCh17G010860 |
| CmoAKT1;1 | CmoCh09G004790 |
| CmoAKT1;2 | CmoCh10G006200 |
| CmoAKT1;3 | CmoCh11G005530 |
| CmoSOS1 | CmoCh04G022490 |
| CmoHKT1;1 | CmoCh10G003830 |
| CmoHKT1;2 | CmoCh10G003840 |
| CmoNHX1 | CmoCh01G011470 |
| CmoNHX2 | CmoCh08G011760 |
| CmoNHX3 | CmoCh10G011690 |
| CmoNHX4 | CmoCh11G013070 |
| CmoNHX5 | CmoCh13G003410 |
| CmoNHX6 | CmoCh13G003420 |
| CmoNHX7 | CmoCh17G011050 |
